# Identification of Synergistic Drug Combinations to Target KRAS-Driven Chemoradioresistant Cancers Utilizing Tumoroid Models of Colorectal Adenocarcinoma and Recurrent Glioblastoma

**DOI:** 10.3389/fonc.2022.840241

**Published:** 2022-05-18

**Authors:** Kshama Gupta, Jeremy C. Jones, Virginea De Araujo Farias, Yuri Mackeyev, Pankaj K. Singh, Alfredo Quiñones-Hinojosa, Sunil Krishnan

**Affiliations:** ^1^ Department of Cancer Biology, Mayo Clinic, Jacksonville, FL, United States; ^2^ Department of Oncology, Mayo Clinic, Jacksonville, FL, United States; ^3^ Department of Neurosurgery, Mayo Clinic, Jacksonville, FL, United States; ^4^ Department of Radiation Oncology, Mayo Clinic, Jacksonville, FL, United States; ^5^ Department of Neuroscience, Mayo Clinic, Jacksonville, FL, United States

**Keywords:** KRAS, DDR1, BCR-ABL1, COAD, GBM, chemoradioresistance, EGFR-ERBB2, Wnt/b-catenin

## Abstract

Treatment resistance is observed in all advanced cancers. Colorectal cancer (CRC) presenting as colorectal adenocarcinoma (COAD) is the second leading cause of cancer deaths worldwide. Multimodality treatment includes surgery, chemotherapy, and targeted therapies with selective utilization of immunotherapy and radiation therapy. Despite the early success of anti-epidermal growth factor receptor (anti-EGFR) therapy, treatment resistance is common and often driven by mutations in APC, KRAS, RAF, and PI3K/mTOR and positive feedback between activated KRAS and WNT effectors. Challenges in the direct targeting of WNT regulators and KRAS have caused alternative actionable targets to gain recent attention. Utilizing an unbiased drug screen, we identified combinatorial targeting of DDR1/BCR-ABL signaling axis with small-molecule inhibitors of EGFR-ERBB2 to be potentially cytotoxic against multicellular spheroids obtained from WNT-activated and KRAS-mutant COAD lines (HCT116, DLD1, and SW480) independent of their KRAS mutation type. Based on the data-driven approach using available patient datasets (The Cancer Genome Atlas (TCGA)), we constructed transcriptomic correlations between gene DDR1, with an expression of genes for EGFR, ERBB2-4, mitogen-activated protein kinase (MAPK) pathway intermediates, BCR, and ABL and genes for cancer stem cell reactivation, cell polarity, and adhesion; we identified a positive association of DDR1 with EGFR, ERBB2, BRAF, SOX9, and VANGL2 in Pan-Cancer. The evaluation of the pathway network using the STRING database and Pathway Commons database revealed DDR1 protein to relay its signaling *via* adaptor proteins (SHC1, GRB2, and SOS1) and BCR axis to contribute to the KRAS-PI3K-AKT signaling cascade, which was confirmed by Western blotting. We further confirmed the cytotoxic potential of our lead combination involving EGFR/ERBB2 inhibitor (lapatinib) with DDR1/BCR-ABL inhibitor (nilotinib) in radioresistant spheroids of HCT116 (COAD) and, in an additional devastating primary cancer model, glioblastoma (GBM). GBMs overexpress DDR1 and share some common genomic features with COAD like EGFR amplification and WNT activation. Moreover, genetic alterations in genes like NF1 make GBMs have an intrinsically high KRAS activity. We show the combination of nilotinib plus lapatinib to exhibit more potent cytotoxic efficacy than either of the drugs administered alone in tumoroids of patient-derived recurrent GBMs. Collectively, our findings suggest that combinatorial targeting of DDR1/BCR-ABL with EGFR-ERBB2 signaling may offer a therapeutic strategy against stem-like KRAS-driven chemoradioresistant tumors of COAD and GBM, widening the window for its applications in mainstream cancer therapeutics.

## 1 Introduction

Chemoradioresistance is a multifaceted challenge in advanced cancers (1). Despite several developments made to improve patient outcomes, drivers of stemness and proliferation continue to be the cause of tumor recurrence and fatality (2, 3). High levels of nuclear β-catenin and activated KRAS are considered the major drivers of cancer stem cell (CSC) expansion and cancer dissemination, the leading cause of chemoradiation therapy (CRT) resistance in various aggressive and recurrent tumors like that of the colon and brain (4–23).

Colorectal cancer (CRC), observed as colorectal adenocarcinoma (COAD), is the third most common cancer worldwide and the second highest in cancer mortality. The 5-year survival rate is 15% in advanced cases, which includes 25% of patients at the point of diagnosis (24, 25). The primary management of CRC includes surgery, chemoradiotherapy, and targeted therapies; however, these treatment strategies continue to evolve due to the emergence of acquired resistance and tumor progression to metastasis (26, 27). Mutations in WNT regulators, such as loss-of-function mutations in adenomatous polyposis coli (APC) gene, are the prime triggers for CRC and activating mutations in KRAS (Kirsten’s rat sarcoma virus oncogene), and its downstream effectors (like PI3K and BRAF) serve as subtype-specific oncogenic drivers. KRAS mutations account for almost 40% of genetic alterations in COAD, and 95% of all KRAS mutant tumors are non-responsive to current treatments (28, 29). Amplification in epidermal growth factor receptor (EGFR) signaling is a common cause of pronounced proliferation, survival, and metastasis in cancers and is one of the prime activators of KRAS (30–32). Cetuximab (anti-EGFR) is used in combination with chemotherapy in refractory CRC disease; however, mutations in KRAS or BRAF lead to aberrant activation of mitogen-activated protein kinase (MAPK) signaling, thereby causing cetuximab resistance with limiting its clinical use to wild-type RAS/RAF tumors (33, 34). Moreover, many metastatic CRC patients harboring wild-type KRAS and BRAF are also non-responders to therapy, highlighting the significance of pathway shunts in acquired treatment resistance (35). Phase I/II trials are ongoing to test the efficacy of small-molecule inhibitors of EGFR with cetuximab in combination to obtain a better treatment outcome (36).

A recent study showed cetuximab-resistant CRC tumors to have mutational hotspots located in the genomic landscapes of receptor tyrosine kinases (EGFR-ERBB2), RAS, and WNT pathways, and ERBB2 (HER2 gene) amplifications are observed in colon cancer (37–40). Activated WNT effectors and KRAS positively collaborate to cause acquired cetuximab resistance (41, 42), and high nuclear beta-catenin levels and mutated KRAS are associated with radioresistance in advanced tumors (15–23). Several WNT inhibitors are undergoing clinical trials; however, an intrinsically high WNT activity in stem cells and normal colon/rectum make direct WNT targeting a challenge (43, 44). Additionally, limitations in the development of KRAS mutation-specific inhibitors and the emergence of complementary pathways have rendered KRAS-driven tumors yet an unbeatable foe (45–50). Clinical trials on targeting signaling intermediates downstream of KRAS in the MAPK pathway (RAS-RAF-MEK-ERK) are underway and include inhibition of BRAF and MEK (mitogen-activated protein/extracellular signal-regulated kinase kinase enzymes MEK1/2). However, these strategies have shown only moderate effects due to the parallel survival pathways that divert the signaling from KRAS-RAF-MEK to KRAS-PIK3, and the occurrence of mutations in regulators of KRAS (51–56). Studies show the potential of targeting phosphatases (such as PTPN11 (SHP2) or downstream targets of PIK3/AKT like mTOR, and research to identify novel action targets is increasingly gaining attention (57–65). For instance, DDR1, discoidin domain receptor 1, a receptor for collagens in the extracellular matrix (ECM) of tumors has recently emerged as a potentially targetable gene against KRAS-mutant lung adenocarcinoma, and DDR1 targeting has also been identified to be beneficial against metastatic colon cancers (66–68). We here performed an unbiased drug screen for signaling pathways documented to be contributors to CRC (61–65), with an aim to identify novel drug combinations with EGFR-small-molecule inhibitors that could offer a therapeutic advantage against KRAS-driven cancers and overcome acquired radioresistance and cetuximab resistance mediated by stem-like (WNT activated) and KRAS mutation phenotype utilizing tumoroid models of COAD.

Glioblastoma (GBM) is a highly advanced brain cancer, which is a WHO grade IV astrocytic tumor with a median survival of 12–14 months and 5-year survival of ~5% (69). GBMs exhibit a high degree of intrinsic stemness, and despite the best available treatments, tumors invariably recur (21–23). While KRAS mutations *per se* are less frequently observed (70, 71), GBM shares some common genomic alterations with CRC, like EGFR amplification and WNT activation, which contribute to tumor progression in both primary and recurrent states (72, 73). Additionally, mutations in genes that contribute to activated KRAS signaling, like neurofibromin-1 (NF1), are observed, which make KRAS signaling a potential target in GBM (22, 73). We here utilized GBM as a second tumor model to validate the efficacy of our prime therapeutic combination identified against highly resilient cancers.

## 2 Materials and Methods

### 2.1 Selection of Small-Molecule Inhibitor Panel

A total of 38 inhibitors were selected based on their ability to target various signaling pathway intermediates (Excel sheet 1, sub-sheet, S1.1, S1.2). A total of 33 inhibitors were used in the initial screening, of which 10 inhibitors were registered as FDA-approved drugs, 9 inhibitors were investigational drugs in clinical trials, and 14 inhibitors were drugs under preclinical testing, or tool compounds. The additional five inhibitors incorporated in this study included three multi-tyrosine kinase inhibitors of the BCR-ABL family, dasatinib, imatinib, and nilotinib (FDA approved), a Src kinase inhibitor KB-SRC-4 (preclinical), and BCR-ABL-specific inhibitor GMB475 (PROTAC compound). All inhibitors were dissolved in sterile dimethyl sulfoxide (DMSO) (Sigma-Aldrich, St. Louis, MO, USA) and stored frozen at −20°C. A concentration of 0.1% DMSO was used as a control.

### 2.2 Cell Culture

Human cell lines HCT116, DLD1, SW480, and CaCo2 (for COAD) and U251 (for GBM) were purchased from the American Type Culture Collection (ATCC) or Sigma-Aldrich and maintained in ATCC-recommended media as per standard cell culture practices. Patient-derived GBM lines were obtained from Quiñones Lab, Neurosurgery, Mayo Clinic (74, 75).

#### 2.2.1 3D Multi-Spheroid Cultures

Multi-spheroid 3D cultures were established by seeding cells at optimized densities between 4,000 and 7,000 cells/well in standard 96-well flat-bottom plates having Nunclon delta surface (167008, Thermo Fisher Scientific, Waltham, MA, USA) in their respective culture media (**Supplementary Figure 1**; **Table 1**). The spheroids were cultivated in media having 1% penicillin/streptomycin (15140122, Gibco, Grand Island, NY, USA), without 10% fetal bovine serum (FBS), and having additional supplements: N2-Max (AR009, R&D Systems, Minneapolis, MN, USA), N21-Max (AR008, R&D Systems), recombinant human EGF (AF-100-15, PeproTech, Cranbury, NJ, USA), recombinant human FGF2 (AF-100-18B, PeproTech), and Insulin-Transferrin-Selenium-Ethanolamine (ITSx, 51500056, Gibco). COAD-spheroid culture media having N2-Max with EGF and FGF2 were defined as N2EF media. Media with N2-Max and N21-Max were defined as N2N21max media, and media having N2-Max plus EGF, FGF2 ( ± N21max), and ITSx were utilized for GBM-multi-spheroid cultures.

**Table 1:** Culture conditions used for multi-spheroid growth.

Tumor type	Cell lines	Media		Medium supplements				
			FBS	N2	N21max	EGF	FGF2	ITSx
COAD	HCT116	DMEM F12	−	+	−	+	+	−
		DMEM F12	−	+	+	−	−	−
COAD	DLD1	RPMI	−	+	+	−	−	−
COAD	SW480	DMEM F12	−	+	+	−	−	−
GBM	U251	DMEM F12	−	+	−	+	+	+
GBM-PD	GBM965	DMEM F12	−	+	+	+	+	+
GBM-PD	QNS108	DMEM F12	−	+	+	+	+	+

Spheroid growth was monitored utilizing Multi-Tumor Spheroid module installed in IncuCyte^®^ Live-Cell Imaging System (IncuCyte SX3, Sartorius, Goettingen, Germany), which can evaluate the label-free development of 3D spheroids in real time. The images obtained were masked or pseudo-colored by the built-in IncuCyte^®^ image processing component. Changes in spheroid total area, spheroid average area, and spheroid eccentricity were plotted over time as measures of spheroid development, growth, and circularity, to obtain a breadth of information on the spheroid formation and health pre- or post-drug treatments. Additionally, averaged spheroid size was estimated in the bright field using Evos FL microscope at 40× magnification (15 spheroids imaged per culture condition and average diameter recorded). Metabolic activity as a measure of spheroid cell viability over time was measured using the luminescence-based cell ATP release assay CellTiter-Glo^®^ (G7572, Promega, Madison, WI, USA). Viable cells were counted at the beginning of every experiment using a TC20 automated cell counter (Bio-Rad, Hercules, CA, USA).

### 2.3 Irradiation

X-ray irradiation (IR) was administered on 24-h cultured multi-spheroids utilizing X-RAD 160 X-Ray Biological Irradiator (Precision X-Ray Inc., North Branford, CT, USA) at max mA = 18.7, max kV = 160, and dose rate 403.8 cGy/min. After IR treatment, the spheroids were continually cultured until two time-points: 24 h post-IR and day 5 post-IR treatment. Spheroid growth and health were evaluated based on the increase in ROS levels (ROS-Glo™ H_2_O_2_ Assay, G8820, Promega), change in spheroid cell viability (CellTiter-Glo^®^, G7572, Promega), and induction of caspase 3/7 activity (Caspase-Glo^®^ 3/7 Assay System, G8090, Promega). All assays were performed as per the manufacturer’s protocol (scheme, **Supplementary Figure 1C**). To evaluate the effect of drugs on overcoming intrinsic radioresistance and spheroid viability post-IR treatment, HCT116 spheroids were cultured for 24 h, IR treated at doses of 4, 8, 12, 16, and 20 Gy; and the respective drugs were administered 24 h post-IR with DMSO as control. The spheroids were continually cultured till day 5 post-IR, and spheroid health was estimated by change in spheroid cell viability and induction of caspase 3/7 activity.

### 2.4 Multi-Spheroid Drug Screens

#### 2.4.1 Single Drug Testing

Exponentially growing HCT116 multi-spheroids cultured in N2EF media were administered with inhibitors serially diluted in Dulbecco’s modified Eagle’s medium (DMEM) (no FBS) at doses up to 25 µM in triplicates. After 2 days post-drug administration, the spheroid viability was measured (CellTiter-Glo, G7572, Promega). Single drug response curves were obtained similarly for the other COAD lines investigated. For GBM line U251, spheroids were obtained by day 6, drug treatments were performed, and measurements were done by day 12. GBM-PD lines GBM965 and QNS108 were evaluated for intrinsic radiosensitivity (**Supplementary Methods**), and inhibitors were administered as single agents or in combination, to monitor their efficacy on spheroid development and growth. Data obtained for each treatment were normalized to DMSO control. Dose–response curves were generated, and IC_50_ values were determined using GraphPad Prism software (GraphPad, Inc., La Jolla, CA, USA).

#### 2.4.2 Combinatorial Drug Testing at Single Dose

To evaluate the drug interactions in a single-dose assay, the drugs identified to have IC_50_ ≤ 25 µM from single drug testing were combined with small-molecule inhibitors for wild-type EGFR (lapatinib, afatinib, and sapitinib), at a combination dose that is less than or equal to their estimated IC_50_ using the experimental scheme (**Supplementary Figure 1C**), unless specified. The treatments were done for 2 days on COAD spheroids, 5 days on GBM (U251) spheroids, and 6 days on GBM-PD lines, and the combinatorial drug response was measured based on spheroid viability assay (CellTiter-Glo, G7572, Promega). All treatments were done in triplicates, and data were normalized to that of DMSO control. Doses used for combination testing are in *Excel sheet 1, sub-sheet 1.2, 1.3*.

#### 2.4.3 Drug Synergy Testing

To identify the interactions between two drugs and to estimate whether the effect was additive, synergistic, or antagonistic, the drugs were combined at 5 individual doses each. These 5 doses for each drug were selected such that they equi-proportionally spread across its estimated IC_50_ and follow the IC_50_ potency ratio as described by Grapsa and Syrigos (49) and Fan et al. (50). In a matrix of 5 doses of drug 1 and 5 doses of drug 2, 5 (drug 1) × 5 (drug 2) = 25 different combinations were obtained and administered in quintuplicates on exponentially growing multi-spheroid cultures at conditions optimized for single-drug testing. For each drug, all 5 doses were also administered as a single agent, along with DMSO as untreated control. The spheroid viability was measured, and data were computed into the MacSynergy-II software (76–78). Peak synergy score was assessed based on the guidelines by MacSynergy-II (at >95% confidence limit) where a synergy score of 0–25 units indicates insignificant synergy; 25–50 units, minor but significant synergy; 50–100 units, moderate synergy; and >100 units, strong synergy. The volume of synergy was determined as per cumulative synergy observed at the 99% confidence limit. Data obtained were represented in the form of i) 3D surface plots, where contours above the plane were indicative of synergy and depressions below the plane indicated antagonism; ii) dot plots, where spheroid viability for all doses of drug 1 and drug 2 was presented against the 25 different combinations administered, with each dot representing an individual treatment condition; and iii) bar graphs, for spheroid viability measurements done at the treatment dosage where synergy score was maximum.

### 2.5 Clonal Cell Proliferation Assay

HCT116 cells were plated at optimized seeding density (600 cells/well) in a standard 96-well plate (167008, Thermo Fisher Scientific), in growth media McCoy’s 5A medium (16600082, Gibco) with 10% FBS and 1% penicillin/streptomycin. Drugs were administered after 24 h, and colony growth was monitored over time utilizing IncuCyte’s built-in clonal dilution module. Percent cell confluence was estimated as a measure of cell proliferation, and automated pseudo-colored images of clonal growth were acquired on day 0, day 3, and day 5 for all treatment conditions. CaCo2 cultures were evaluated similarly for clonal cell proliferation (**Supplementary Figure 7B**). To assess clonal cell growth and proliferation over time in patient-derived GBM lines (PD-GBM) GBM965 and QNS108, the cells were seeded at a density of 1,000 cells/well in a standard 96-well plate in stem-cell media (same as utilized for spheroid cultures, having DMEM-F12, supplemented with N2-Max plus, N21max, EGF, FGF2, and ITSx), with or without the presence of drug combinations being tested. The cells were allowed to grow for a week and then assessed for viability (CellTiter-Glo^®^, G7572, Promega).

### 2.6 Cancer Genomics

i) *Evaluation of percent genetic variations:* COAD and GBM datasets from The Cancer Genome Atlas (TCGA) combined with all COAD or GBM studies deposited at the cBioPortal database and Pan-Cancer Atlas were utilized to obtain percent genetic alterations for the selected gene list (source: https://www.cbioportal.org/). ii) *Relative gene expression:* The mRNA expression of the selected genes was evaluated and compared in two biological states: normal and tumor, for both COAD and GBM, utilizing gene expression profiling interactive analysis, GEPIA (http://gepia.cancer-pku.cn/), which links the datasets from TCGA (for tumor) and GTEx (for normal). iii) *Profiling of genes co-expressed with DDR1 and KRAS:* GEPIA was utilized to perform a pairwise gene expression correlation analysis of genes DDR1 and KRAS with selective genes through TCGA and GTEx expression datasets using Pearson’s method. Additionally, the Pan-Cancer Atlas datasets (c-Bioportal) were utilized to perform a pairwise gene expression correlation analysis using Pearson’s and Spearman’s methods. iv) Protein–protein interactions were evaluated utilizing the BioGRID (https://thebiogrid.org/) and STRING (https://string-db.org/) databases, and pathway engagement was further assessed using Pathway Commons (https://www.pathwaycommons.org/). v) IC_50_ correlations for EGFR inhibitors and BCR-ABL1 inhibitors were obtained for COREAD and Pan-Cancer cell line datasets using the Genomics of Drug Sensitivity in Cancer (*GDSC*) database (https://www.cancerrxgene.org/).

### 2.7 Western Blotting

Western blotting was performed as per standard protocol. Antibodies used were purchased from Cell Signaling Technology (Danvers, MA, USA). These include the following: Phospho-Bcr (Tyr177) (#3901), BCR (#3902S), Phospho-Akt (Ser473) (D9E) XP (#4060), Akt1 (D9R8K) (#75692), Phospho-p44/42 MAPK (Erk1/2) (Thr202/Tyr204) (D13.14.4E) XP (#4370), c-ABL1 (#2862S), DDR1 (D1G6) XP (#5583), β-Catenin (D10A8) XP (#8480), α-Actinin (D6F6) XP (#6487), and GAPDH (D16H11) XP (#5174). The secondary antibody used was anti-rabbit IgG, horseradish peroxidase (HRP)-linked (#7074). The primary antibodies used in Western blotting were diluted in 1:1,000 or 1:2,000; the secondary antibody was diluted in 1:5,000. All antibodies were previously validated and documented in published literature.

### 2.8 Statistical Analysis

Data analysis was performed using GraphPad Prism, Student’s t-test was utilized to compare sample means, and statistical significance was indicated as *p < 0.05, **p < 0.01, ***p < 0.001, and ****p < 0.0001. All data are presented as the mean of at least two independent experiments with error bars as SEM.

## 3 Results

### 3.1 Growth and Expansion of Multi-Spheroids in Defined Media

**Figure 1:**
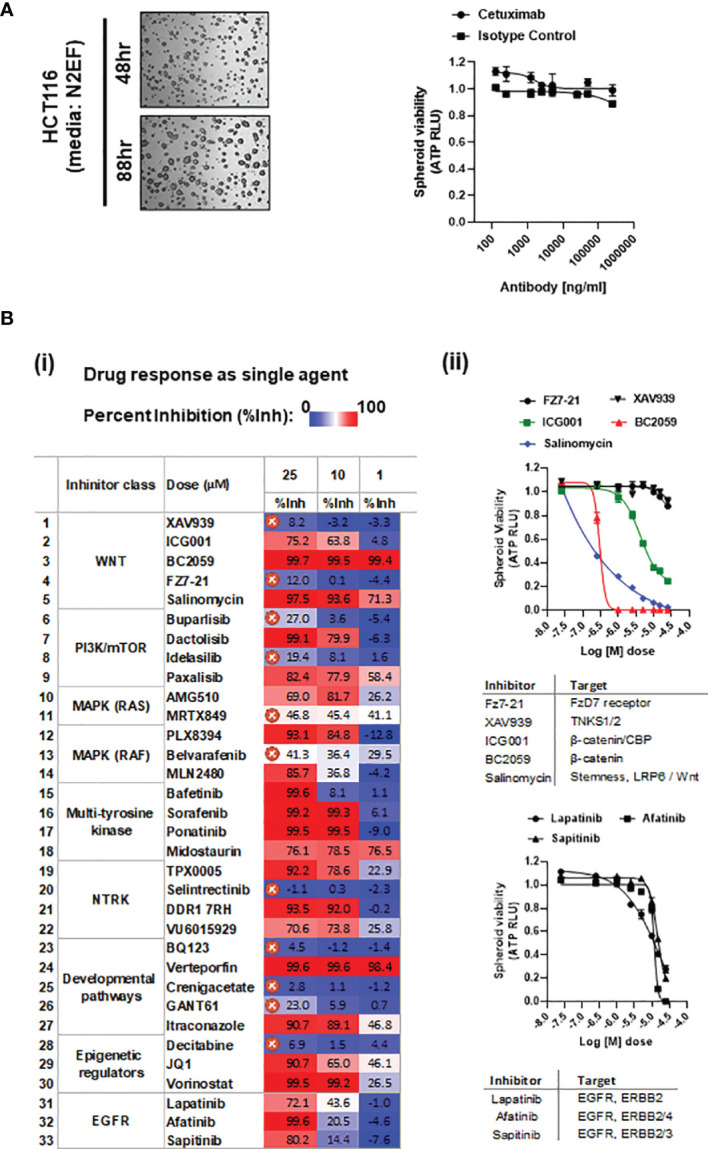
**(A)** Representative bright-field images of HCT116 spheroids cultured in media having nitrogen supplement (N2), plus EGF and FGF2 (N2EF), taken at time-points indicated. **(i)** Spheroid viability in response to cetuximab (anti-EGFR) treatment on HCT116 multi-spheroids cultured in N2EF media. **(B) (i)** Drug screening: inhibitors listed were administered as single agents on HCT116 (N2EF) multi-spheroids, and spheroid viability was estimated. Table includes percent inhibition (%Inh.) observed for each drug when administered at concentrations of 25, 10, and 1 µM (dose–response curves are included in **Supplementary Figure 3**). IC_50_ was not reached for drugs marked with a cross, which were excluded further from the study. **(ii)** Single-agent spheroid viability curves for WNT pathway inhibitors and small-molecule inhibitors to EGFR/ERBB proteins on HCT116 multi-spheroids.

Multi-spheroid cultures for all cell lines utilized in this study were optimized and established as described in the Methods section, and culture conditions for each line are included in **Table 1** and **Supplementary Figure 2**. HCT116 multi-spheroid showed comparable growth in both culture medium compositions tested, N2EF and N2N21max (**Figure 1A**; **Supplementary Figure 2**). Thus, all experiments were carried out in N2EF media for HCT116, unless otherwise indicated. DLD1 formed proliferative spheroids only in N2N21max-supplemented media (**Supplementary Figure 2**). The exponential growth of spheroids was observed between day 1.5 and day 4.5 for both the lines [**Supplementary Figure 2C**(**ii**)]. Therefore, all drug screens were performed within this period. Spheroid culture parameters evaluated for SW480 showed a similar trend [**Supplementary Figure 2C**(**iii**)]. GBM lines were slower in growth relative to COAD lines investigated. Drug treatments in U251 cells were therefore performed between day 6 and day 12. Spheroid cultures for PD-GBM lines GBM965 and QNS108 were established likewise, drugs were administered on day 6, and viability was measured by day 8 in culture.

### 3.2 Treatment Resistance in Multi-Spheroids of Colorectal Adenocarcinoma

#### 3.2.1 HCT116 Multi-Spheroids Exhibit Intrinsic Resistance to Cetuximab and Irradiation Treatment

Cetuximab administered up to a final concentration of 250 µg/ml on HCT116 multi-spheroids showed no cytotoxic effect, indicating anti-EGFR resistance in HCT116 (**Figure 1A** and **Supplementary Figure 2D**). Studies have reported abrogation of RAS signaling as one of the mechanisms to enhance radiosensitivity in HCT116, indicating a certain degree of resistance to radiation treatment in KRAS mutant lines (79, 80). To evaluate the effect of IR on spheroid survival and growth, HCT116 multi-spheroids were administered with single-dose radiations of 0, 4, 8, 12, 16, and 20 Gy, and spheroid viability was compared between two time-points, 24 h and day 5 post-IR (scheme, **Supplementary Figure 1C**). Irradiated spheroids showed a potential decline in spheroid growth from 0 to 20 Gy at both time-points, which was expected since 20 Gy is a high-enough single dose to be administered *in vitro*. However, the spheroids that were cultured till day 5 post-IR also showed significantly more viability at all IR treatments as compared to viability observed at each of these respective doses at 24 h post-IR. This indicated that while single-dose treatments were sufficient to kill a large number of cells in spheroid cultures, some cells were able to resist or recover from radiation-induced stress and led to the re-establishment of spheroids by day 5. Increased spheroid viability at day 5 post-IR as compared to 24 h post-IR is indicated (p-value < 0.0001) (**Supplementary Figure 2D**).

### 3.3 Small-Molecule Inhibitor-Based Drug Screening in HCT116 Multi-Spheroids

#### 3.3.1 21 out of 33 Inhibitors Showed Cytotoxicity as Single Agent

A drug screen performed on multi-spheroids from HCT116 utilizing a panel of 33 small-molecule inhibitors (*Excel Sheet 1_sub-sheet S1.1*) revealed 21 effective inhibitors as single agents. These included three small-molecule inhibitors of EGFR (lapatinib, afatinib, sapitinib), and the rest of the 19 inhibitors targeting various pathways, like WNT/β-catenin, PIK3/mTOR dual kinase, epigenetic regulators, RAF/BRAF, multi-tyrosine kinases, and NTRK (neurotrophic receptor tyrosine kinases). Challenges to obtaining KRAS mutation-specific drugs led to a poor response from direct KRAS targeting. Significant cytotoxicity in response to targeting β-catenin or β-catenin/P300 complex (using drugs BC2059 and PRI724) indicated constitutive activation of downstream WNT effectors in HCT116 multi-spheroids [**Figure 1B**(**ii**)]. A color-scaled tabulated comparison of percent inhibition values observed for individual drug treatment at doses 25, 10, and 1 µM reveals the relative potency of each of these inhibitors. The inhibitors for which IC_50_ was not reached at a concentration of 25 µM were excluded from the study [**Figure 1B**(**i**)]. Dose–response curves are included in **Figure 1B**(**ii**) and **Supplementary Figure 3**. Inhibitory dose 50 (IC_50_) values estimated are included in *Excel Sheet 1, sub-sheet S1.2*.

### 3.4 Combinatorial Drug Testing With EGFR Inhibitors in Colorectal Adenocarcinoma Multi-Spheroids

#### 3.4.1 Top 5 Leads Identified From Combinatorial Drug Testing at Single Dose

**Figure 2:**
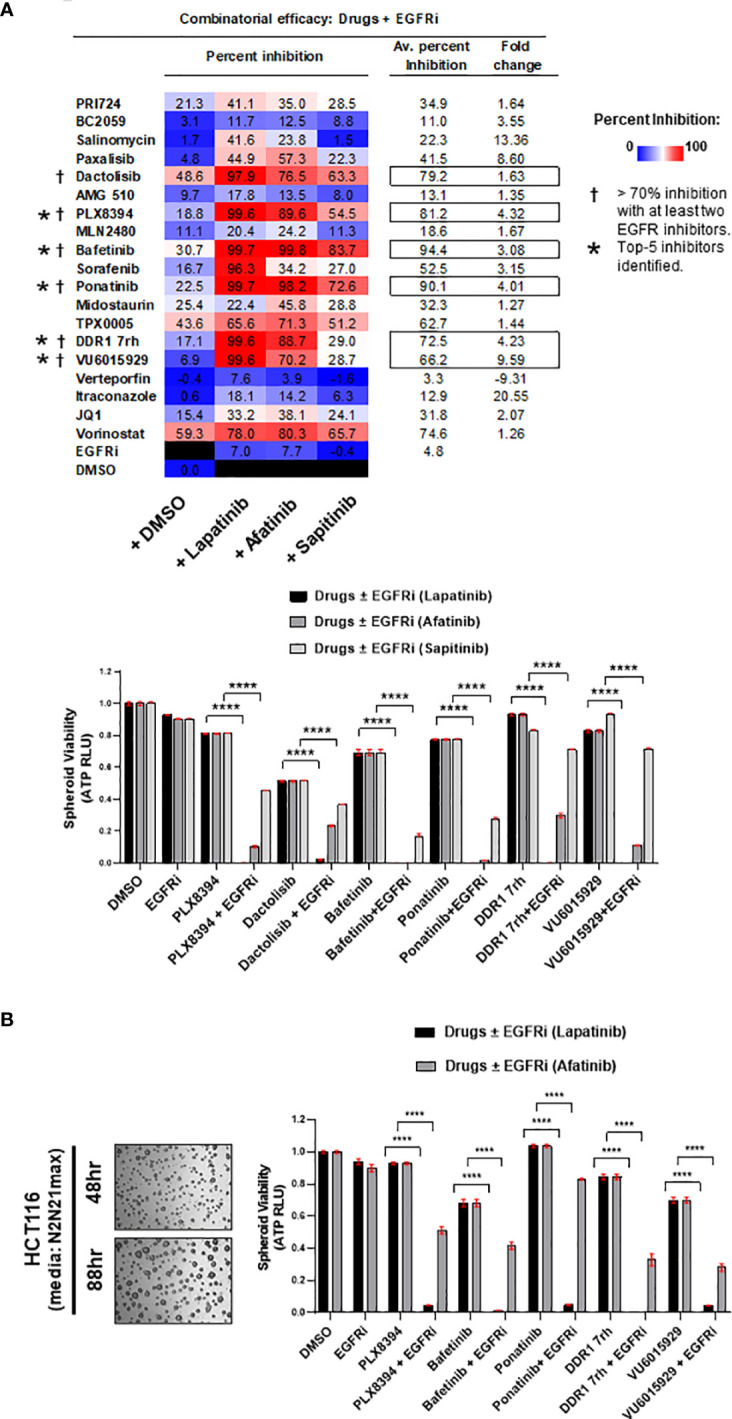
**(A)** Efficacy of the drugs in combination with EGFR small-molecule inhibitors (EGFRi). Drugs were administered on multi-spheroid of HCT116 (N2EF), and spheroid viability was measured. Table indicates percent inhibition obtained for each of the drugs alone, or in combination with lapatinib, afatinib, or sapitinib. Color scale of percent inhibition indicates overall combinatorial efficacy of lapatinib > afatinib > sapitinib. In columns on the right, averaged percent inhibition for each drug when administered in combination with all three EGFRi, and fold change between drug+EGFRi and drug alone is mentioned. Inhibitors marked with a dagger (†) are prime 6 combinatorial drugs identified, which showed >70% inhibition with at least two out of three EGFR inhibitors tested. Top 5 inhibitors (marked with asterisk, *) had averaged percent inhibition ≥50 and fold change ≥2. Bar graphs indicate relative spheroid viability of HCT116 multi-spheroids when administered with the prime 6 combinatorial leads identified. **(B)** Representative images of HCT116 multi-spheroids obtained by culturing in additional media supplemented with N2 and N21max. Top 5 combinatorial leads identified were validated in combination with EGFR inhibitors (lapatinib and afatinib) on HCT116 multi-spheroids cultured in N2N21max. Bar graphs for combination treatments tested are represented on the right. ****p < 0.0001.

The 19 inhibitors that were identified to be effective as single agents were evaluated for their efficacy in combination with small-molecule inhibitors for wild-type EGFR (EGFRi: lapatinib, afatinib, and sapitinib) on multi-spheroids of HCT116. This revealed 9 inhibitors, which exhibited enhanced cytotoxicity in combination, which were paxalisib, dactolisib, PLX8394, bafetinib, ponatinib, TPX0005, DDR1 7rh, VU6015929, and vorinostat (**Supplementary Figure 4A**). Sorafenib showed a promising response only with lapatinib, and the remaining inhibitors showed moderate-to-low effect in combination with EGFRi (**Supplementary Figure 4B**). The prime 6 leads were considered the combinations where percent inhibition obtained with at least two out of three EGFRi was >70%. The top 5 leads were identified based on two criteria: a) if % inhibition obtained in combination with at least two out of three EGFRi was ≥50% and b) if the fold change between drugs+EGFRi and drug alone was ≥2. The drugs that met both these criteria were bafetinib, ponatinib, VU6015929, DDR1 7rh, and PLX8394 (**Figure 2A**). The overall combinatorial response for all 3 EGFRi was in the order lapatinib > afatinib > sapitinib; therefore, sapitinib was excluded from further validations (**Figure 2A**, *Excel sheet 1, sub-sheet S1.4*). The top 5 candidates were further validated for their efficacy in combination with EGFRi lapatinib and afatinib, when administered on HCT116 multi-spheroids cultured in N2N21max media; and the inhibition obtained was higher with lapatinib (**Figure 2B**).

**Figure 3:**
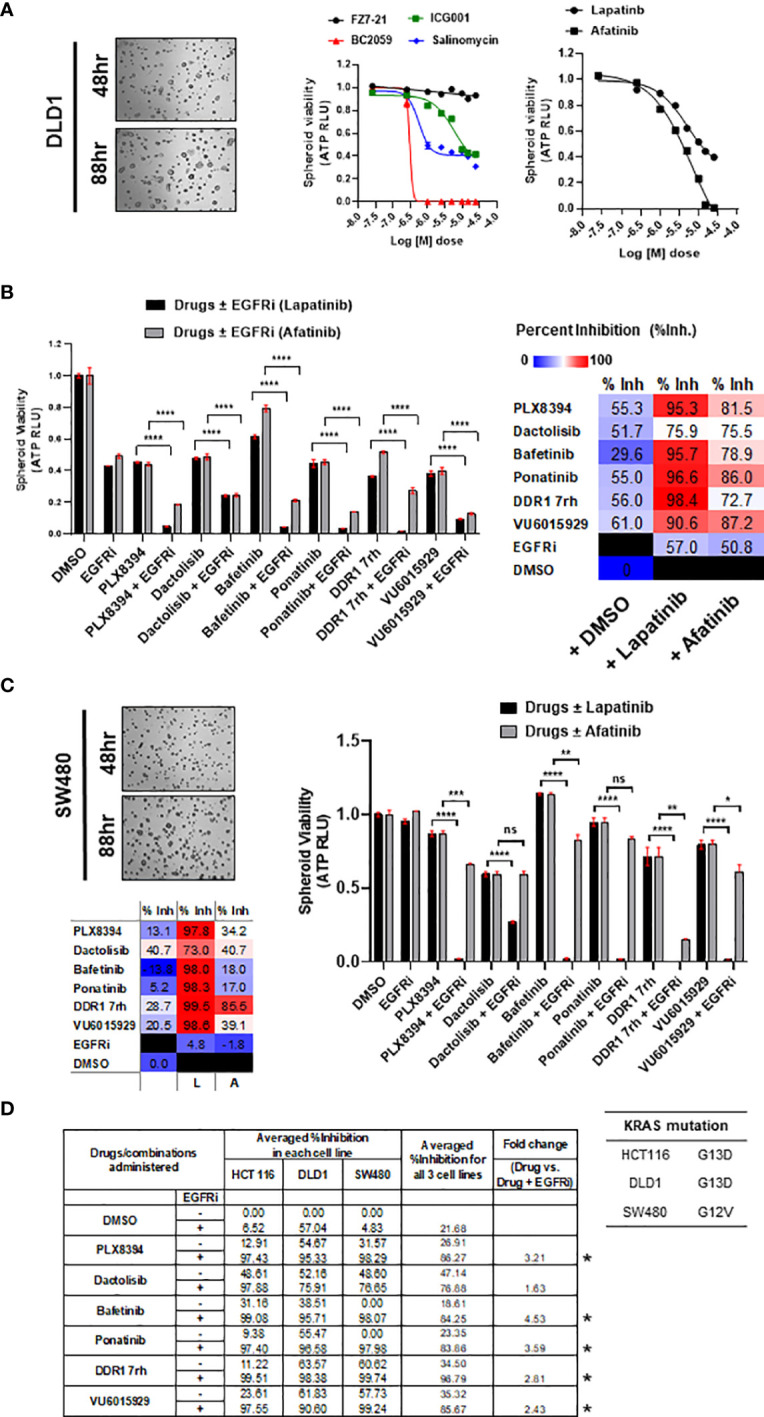
**(A)** Representative bright-field images of multi-spheroids obtained for DLD1 cultured in N2N21max media, at time-points indicated. Dose–response curves (right) indicate the efficacy of WNT pathway inhibitors and EGFR small-molecule inhibitors (lapatinib, and afatinib) as single agents on DLD1 spheroid viability. **(B)** Combinatorial efficacy of the prime leads identified when administered on multi-spheroids from DLD1. Bar graphs indicate relative spheroid viability of DLD1 when treated with respective drugs ± EGFR inhibitors (lapatinib and afatinib). Table on the right shows percent inhibition values calculated for all 6 drugs alone, or in combination with EGFRi lapatinib and afatinib. Color scale indicates lapatinib to have higher combinatorial efficacy than afatinib. **(C)** Combinatorial efficacy of lapatinib with prime 6 leads was validated in an additional line SW480, harboring KRAS G12V mutation. Representative bright-field images (left) show multi-spheroids for SW480 cultured in N2N21max media at indicated time-points. Table lists KRAS mutations known for the respective cell line SW480, having G12V, as opposed to G13D in HCT116 and DLD1. Bar graphs (right) indicate relative spheroid viability of SW480 when administered with respective drugs ± EGFR inhibitor lapatinib. **(D)** Table (bottom) includes averaged percent inhibition obtained for each of the COAD cell lines (HCT116, DLD1, and SW480) when administered with drugs ± EGFR inhibitor, lapatinib, and afatinib. Averaged percent inhibition for all three cell lines, and fold change observed between drug administered in combination (+EGFRi) and drug alone is mentioned in columns on the right. Inhibitors marked with an asterisk are the top 5 combinatorial inhibitors identified. *p < 0.05, **p < 0.01, ***p <0.001, and ****p < 0.0001.

DLD1, a cell line classified to be stem-like and known to be heterozygous KRAS^G13D^ mutation, was confirmed for its intrinsically high WNT activity by its response to inhibition of WNT effector, β-catenin (**Figure 3A**). The prime 6 leads when administered in combination with EGFR inhibitors on DLD1 multi-spheroids showed a similar trend as observed for HCT116. Percent inhibition obtained in combinations with lapatinib was greater than afatinib (color-scaled table, **Figure 3B**). To test if these observations were KRAS mutation-type independent, the combinatorial efficacy of drugs ± EGFR inhibitor (lapatinib or afatinib) was validated in spheroid cultures of SW480, a COAD cell line homozygous for KRAS^G12V^ mutation (**Figure 3C**). The top 5 leads when administered in combination with EGFRi (lapatinib and afatinib) showed ≥95% averaged percent inhibition for all three COAD lines tested and had an overall fold change ≥2 (**Figure 3D**). Bafetinib and ponatinib showed the best combinatorial response in all 3 lines. Drug doses used for each of the combinatorial drug tests are included in *Excel sheet 1, sub-sheet S1.3*.

#### 3.4.2 Top 5 Combinatorial Leads Synergize With EGFR Inhibitor, Lapatinib

**Figure 4:**
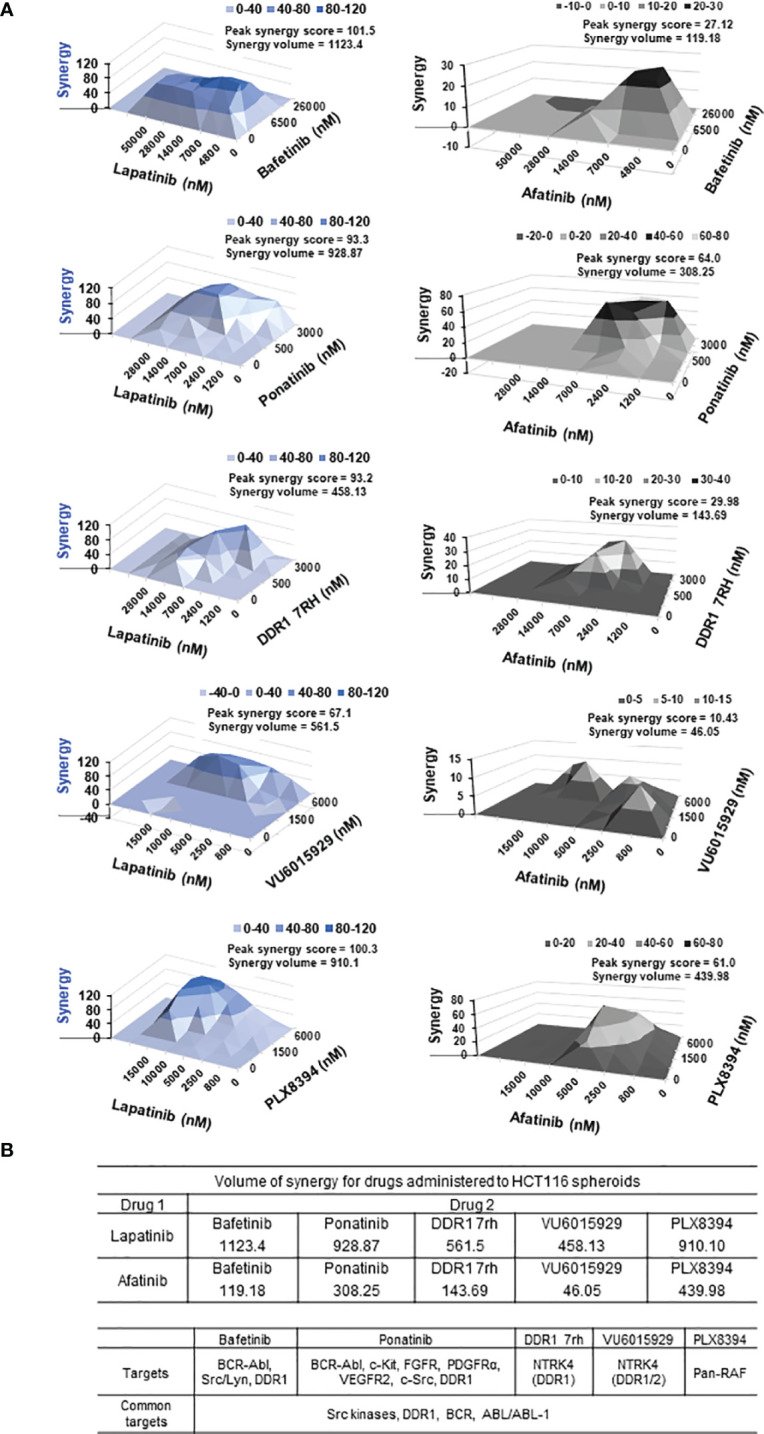
**(A)** Synergy plots for EGFR inhibitors lapatinib (left) and afatinib (right), when administered in combination with drugs (top 5 combinatorial leads: bafetinib, ponatinib, DDR1 7rh, VU6015929, and PLX8394) on HCT116 multi-spheroids cultured in N2EF media. 3D surface plot having a rise of the curve in positive xy-axis indicates synergy. Peak synergy score and volume of synergy (cumulative synergy score) obtained for each of the combinations are indicated in the top right corner of surface plot. Synergy plots were made at the 99% confidence limit. Table (bottom) shows comparison of cumulative synergy scores (volume of synergy), obtained for drug 1 (EGFR inhibitors lapatinib, or afatinib) when combined with drug 2. Details on synergy or antagonism score obtained and dose at which peak synergy score was observed for each of the combinations are tabulated in **Supplementary Figure 5**. A matrix for synergy score values and a matrix for percent inhibition obtained for all 25 combination treatments were been created for each of the synergy experiments performed and included in **Supplementary Figure 5**. **(B)** Table lists targets for top 5 combinatorial leads and targets common among them. DDR1, discoidin domain receptor 1 (neurotrophic receptor tyrosine kinase, NTRK4), came up as the most prevalent target among the combinatorial leads.

Synergy was identified for the prime 5 leads (PLX8394, bafetinib, ponatinib, VU6015929, DDR1 7rh) in combination with both EGFRi lapatinib and afatinib (**Figure 4**, **Table 2**). When administered in combination with lapatinib, drugs that showed peak synergy score ≥100 (indicating strong synergy) were bafetinib and PLX8394, and those within 50–100 (indicating moderate but significant synergy) were ponatinib, DDR1 7rh, and VU6015929. When administered in combination with afatinib, none of the drugs showed a peak synergy score ≥100, and the only drugs that showed peak synergy between 50 and 100 were ponatinib and PLX8394. A widespread rise in 3D surface plot observed for lapatinib plus bafetinib indicated it to have the highest synergy volume (1,123.4). Comparing the values of volume of synergy obtained for all drugs, combinations with lapatinib showed an overall higher synergy and in the order bafetinib > PLX8394 = ponatinib > DDR1 7rh > VU6015929. Thus, EGFR/ERBB2 inhibitor lapatinib was identified to be a more promising combinatorial drug in COAD. The matrix showing synergy scores and percent inhibitions obtained at each of the individual 25 combinations administered per synergy evaluation for all 5 drugs (in combination with lapatinib and afatinib) are included in **Supplementary Figures 5A, B** (dot plots and bar graphs for spheroid viability are in **Supplementary Figure 5C**).

**Table 2:** Synergy scores obtained in HCT116 multi-spheroid viability assay.

Drug 1	Drug 2	Cumulative scores obtained		Max. synergy obtained at		
		Synergy score	Antagonism	Drug 1	Drug 2	Synergy score
Lapatinib	Bafetinib	1,123.4	0	7µM	6.5µM	101.45
	Ponatinib	928.87	0	7µM	3µM	93.3
	DDR1 7rh	561.5	0	2.4µM	3µM	93.2
	VU6015929	458.13	−12.44	10µM	6µM	78.78
	PLX8394	910.15	0	15µM	6µM	100.3
Afatinib	Bafetinib	119.18	−0.29	4.8µM	13µM	27.13
	Ponatinib	308.25	−0.02	2.4µM	3µM	64.04
	DDR1 7rh	143.69	0	2.4µM	3µM	30
	VU6015929	46.05	0	10µM	6µM	10.43
	PLX8394	439.98	0	5µM	3µM	61.06

#### 3.4.3 DDR1, a Common Target Among Synergistic Leads Identified

To address why these 5 inhibitors selectively emerged as potential synergistic leads, we looked at their common targets. These were found to be as follows: 1) DDR1 (discoidin domain receptor 1), 2) BCR-ABL kinases, and 3) Src kinases (Src/Lyn) (**Figure 4B**). Bafetinib and ponatinib belong to the BCR-ABL family of multi-tyrosine kinase inhibitors, which also target DDR1 (81, 82), and DDR1 was a common target among 4 out of the top 5 combinatorial leads.

### 3.5 Cancer Genomics

To investigate the signaling cross-talks among targets of combinatorial drugs (viz., PLX8394, bafetinib, ponatinib, DDR1 7rh, VU6015929, lapatinib, and afatinib), we utilized a genomics approach. A panel of 35 genes was selected incorporating their direct or indirect targets, and related biological processes (cell adhesion, proliferation, migration, and stemness). Since DDR1 is the most prevalent target among synergistic drugs identified and is known to be highly elevated in tumors of the brain, we performed a parallel comparison of our selected gene panel for percent genomic alterations, relative mRNA expression, and transcriptional correlations in patient datasets of COAD, GBM, and Pan-Cancer Atlas (source, genomics portals: cBioPortal and GEPIA). Transcriptional correlations were also made with KRAS to identify common associations between DDR1 and KRAS signaling.

#### 3.5.1 DDR1 Positively Correlates With BCR, EGFR, ERBB2 in Pan-Cancer

**Figure 5:**
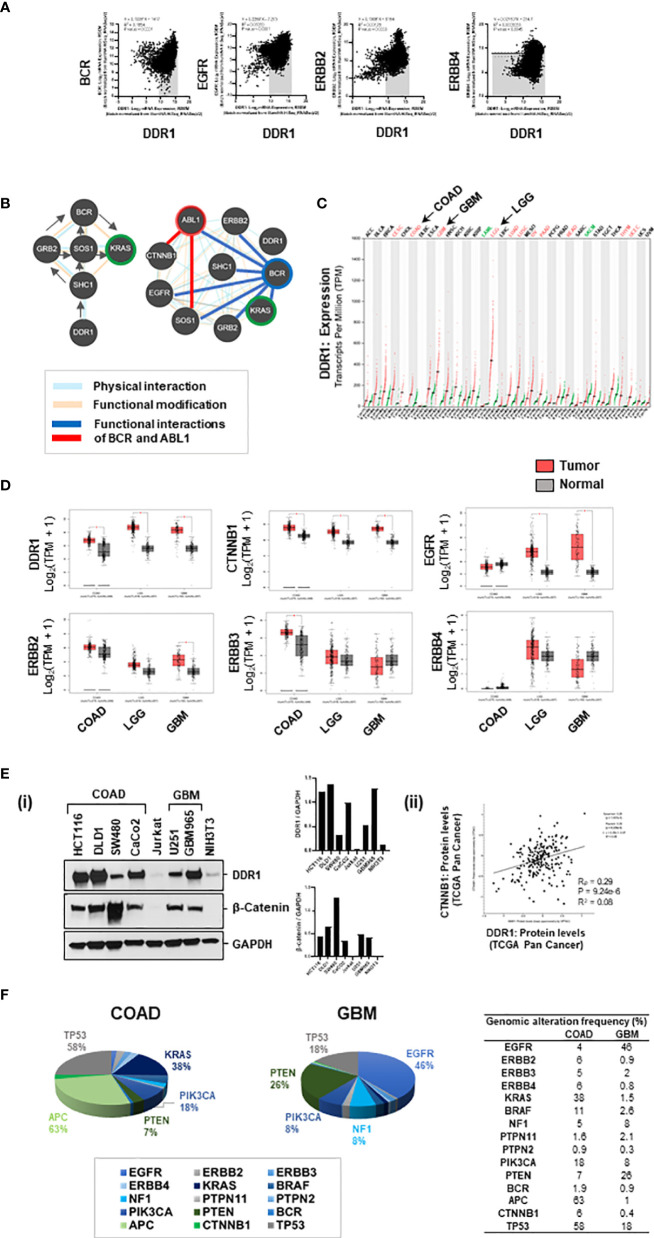
**(A)** Genomic associations of DDR1 and EGFR/ERBB2: DDR1 gene positively correlates with genes BCR, EGFR, and ERBB2 in Pan-Cancer (source: cBioportal). **(B)** Based on pathway commons database, the signaling relay from DDR1 to BCR and KRAS engages adaptor proteins SHC1, GRB2, and SOS1. The protein interactome of DDR1, BCR, and ABL1 with selective proteins on the right shows physical interactions of DDR1 with ERBB2 and SHC1; physical and functional interactions of BCR with EGFR, ERBB2, adaptors, KRAS, and ABL1; and functional interactions of ABL1 with SOS1 and CTNNB1. **(C)** Relative expression of DDR1 gene in datasets of tumor versus normal samples for various cancer subtypes revealed DDR1 to be maximally elevated in brain cancers, low-grade gliomas (LGG), and glioblastoma (GBM). **(D)** Boxplots showing relative expression of genes DDR1, CTNNB1 (beta-catenin), EGFR, ERBB2, ERBB3, and ERBB4 in patient versus normal datasets for COAD, LGG, and GBM. GBM tumors have elevated expression of DDR1, CTNNB1, EGFR, and ERBB2. **(E) (i)** Western blotting for DDR1 and beta-catenin proteins in spheroid lysates from various cell lines of COAD and GBM show DDR1 to be highly expressed in COAD lines HCT116 and DLD1 and patient-derived GBM line GBM965. Beta-catenin is highly expressed in COAD line SW480 and moderately expressed in GBM lines U251 and GBM965. **(ii)** An overall positive correlation (R = 0.28) was observed between protein expression levels of DDR1 and beta-catenin (CTNNB1) in Pan-Cancer (source: c-Bioportal). **(F)** Percent genomic alterations of selective genes in datasets of COAD and GBM reveal mutations in KRAS and APC to be prime drivers of COAD; and high prevalence of genomic alterations in EGFR and NF1 genes makes KRAS a potential driver of GBM.

Utilizing the patient datasets from Pan-Cancer (c-Bioportal), we observed a significant positive correlation between DDR1 expression and expression of genes BCR, EGFR, and ERBB2 (**Figure 5A**). Additionally, correlation analysis was performed to evaluate associations between the mRNA expression of a selected panel of genes, with mRNA expression of DDR1 and KRAS in patient datasets of COAD, GBM, and Pan-Cancer revealed i) DDR1 to positively correlate with downstream mediators of KRAS signaling (BRAF, PIK3CA, and MTOR) in both tumors COAD and GBM and with EGFR, ERBB2, BRAF, BCR, SOX9, VANGL2, and CDH1 in Pan-Cancer; and ii) KRAS to correlate with genes BRAF, PIK3CA, APC, and CTNNB1 in Pan-Cancer (**Supplementary Figure 6A**) (*Excel sheet 2, sub-sheet S2.3–S2.5*). BRAF, PIK3CA, MTOR, and ABL1 correlated with both DDR1 and KRAS in COAD, indicating the implications of the DDR1-BCR axis in the activation of KRAS and PI3K/mTOR pathway. All correlation coefficients (Pearson, R_p_, or Spearman, R_s_) >0.2 were significant. To further investigate the association of DDR1 with SRC/BCR-ABL signaling in COAD, we generated a correlation matrix with selective genes. Significant correlations were observed between the expression of DDR1, EGFR, and SRC/BCR axis, indicating its active involvement in COAD [source: GEPIA; datasets, TCGA (tumor), GTEx (Normal)] (**Supplementary Figure 6B**).

#### 3.5.2 DDR1 Signaling Relay to BCR and KRAS

To investigate the interconnections of DDR1, BCR, and ABL1 with KRAS signaling, SRC kinases (SRC/LYN), WNT effector (CTNNB1), and EGFR signaling (EGFR, ERBB proteins), we performed protein–protein interaction analysis using BioGRID, STRING, and Pathway Commons databases. ERBB2 and CDH1 were found as close associates of DDR1 in BioGRID (*Excel sheet 2, sub-sheet S2.6–S2.7*). STRING protein interactome was generated for known physical interactions between 32 proteins (32 nodes and 112 edges) using MCL clustering (inflation number 5), and 8 clusters were revealed with high confidence (score >0.7) (**Supplementary Figure 6C**). The network obtained showed i) the adaptor protein SHC1 interacts with DDR1, ii) KRAS interacts with PI3K/AKT, iii) GRB2 and SOS1 connect SHC1 with BCR and ABL1, and iv) BCR interacts with ABL1 (independent of gene fusions) and ABL1 complexes with CTNNB1. This was confirmed by physical and functional interactions identified based on the Pathway Commons database, which also revealed signaling relay from BCR to KRAS *via* protein adaptors GRB2 and SOS1 (**Figure 5B**). Together, this indicates signaling relay from DDR1 (as homodimer or heterodimer with EGFR/ERBB2) *via* SHC1, GRB2, SOS1 to BCR-ABL1 complex, and from BCR-ABL1 to KRAS and PI3K/AKT. The details of protein interactome are included in *Excel sheet 2 (sub-sheet S2.6–S2.8)*.

#### 3.5.3 Elevated Expression of DDR1 in Colorectal Adenocarcinoma and Glioblastoma

Comparing the expression of DDR1 gene in various tumor models revealed DDR1 to be elevated in all tumors with maximal expression in brain cancers, low-grade gliomas (LGG), and GBM (**Figure 5C**) [source: GEPIA; datasets, TCGA (tumor), GTEx (Normal)]. Comparing the relative expression of selective genes between COAD, LGG, and GBM, we observed DDR1 and beta-catenin (CTNNB1) genes to be elevated in all three tumor types. Moreover, genes EGFR and ERBB2 were highly elevated in GBMs, making GBM a perfect model for revalidation of our prime synergistic combination (**Figure 5D**). Looking at the relative expression of DDR1 and beta-catenin proteins in cell lines of COAD and GBM, we found HCT116, DLD1, and GBM965 to be overexpressing DDR1 [**Figure 5E**(**i**)]. Moreover, an overall positive correlation (R = 0.28) was observed between protein expression levels of DDR1 and beta-catenin (CTNNB1) in Pan-Cancer (source: c-Bioportal) (**Figure 5E**(**ii**), **Supplementary Figure 6E**).

#### 3.5.4 KRAS, a Tumor Driver in Colorectal Adenocarcinoma and Glioblastoma

Percent genomic alterations in COAD revealed a mutation frequency of 63% in APC gene and 38% in KRAS, making it as expected a highly WNT-activated and KRAS-driven cancer. Moreover, high mutation frequencies in PIK3CA (18%) and PTEN (7%) indicate targeting PI3K/AKT pathway as a potential therapeutic approach. GBM on the other hand showed a very high genomic alteration frequency in EGFR (46%) and negative regulators of KRAS and PI3K, NF1 (8%), and PTEN (26%), making KRAS and its downstream effector PI3K/AKT a targetable driver of GBM (**Figure 5F**) (*Excel Sheet 2, sub-sheet S2.1*).

### 3.6 Targeting DDR1/BCR-ABL With EGFR-ERBB2 in Multi-Spheroids of Colorectal Adenocarcinoma and Glioblastoma

#### 3.6.1 Combinatorial Efficacy of DDR1/BCR-ABL1 Multi-Tyrosine Kinase Inhibitors With Lapatinib

**Figure 6:**
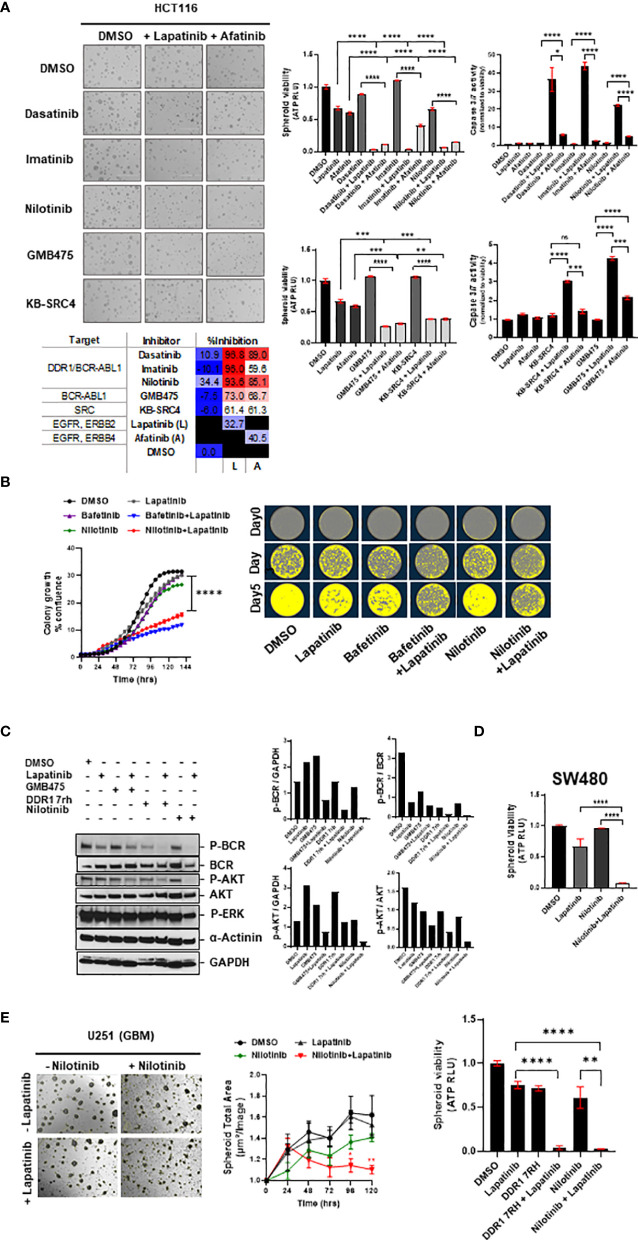
**(A)** Combinatorial efficacy of DDR1/BCR-ABL1 multi-tyrosine kinase inhibitors (dasatinib, imatinib, and nilotinib) and compound drugs GMB475 and KB-SRC4 when administered with EGFRi (lapatinib and afatinib) on multi-spheroids of HCT116. Representative images (left) and bar graphs (right) indicate spheroid viability and relative caspase 3/7 activity (normalized to viability), with percent inhibition listed in the table at the bottom. **(B)** Clonal cell proliferation assay performed for HCT116 cells for drugs bafetinib and nilotinib in combination with lapatinib validated the combinatorial efficacy. Clonal cell growth was measured as percent confluence, using IncuCyte. Representative images (right) for clonal cell proliferation obtained at time-points day 0, day 3, and day 5 are pseudo-colored utilizing IncuCyte’s inbuilt clonal dilution module. **(C)** Western blotting for HCT116 spheroid-lysates prepared 48 h after administration of drugs indicated significant downregulation of phospho-BCR and phospho-AKT levels in DDR1/BCR-ABL with EGFR/ERBB2 inhibitor combination treatment as compared with either of the drugs administered alone. Change in phospho-ERK levels was minimal, indicating the prime signaling affected in combination treatments is BCR-AKT axis. **(D)** Efficacy of nilotinib in combination with lapatinib validated on viability of multi-spheroids from SW480 (KRAS^G12V^). **(E)** Representative bright-field images of U251 (GBM) spheroids treated with drugs lapatinib and nilotinib at day 5 post-treatment. Graph (middle) shows reduction in spheroid total area over 5 days post-treatment. Bar graph (right) indicates loss of spheroid cell viability measured on day 5. Statistical significance is indicated as *p < 0.05, **p < 0.01, ***p < 0.001, and ****p < 0.0001.

To test whether the enhanced combinatorial efficacy observed with bafetinib and ponatinib, when administered in combination with EGFR inhibitor lapatinib, could be extrapolated to also other members of the DDR1/BCR-ABL inhibitor family, we incorporated the drugs dasatinib, imatinib, and nilotinib in our evaluation. We observed nilotinib to be more potent than dasatinib and imatinib as a single agent, and a significant reduction in HCT116 spheroid viability was obtained with all three inhibitors when administered in combination with lapatinib (L) or afatinib (A) (**Figure 6A**). To identify whether combinatorial targeting of EGFR-ERBB2/4 with Src-specific or BCR-ABL1 specific inhibitors could have a similar outcome, two compounds were tested, KB SRC-4 (Src inhibitor) and GMB475 (PROTAC degrader of BCR-ABL). Both compounds showed enhanced response in combination, and BCR-ABL-specific targeting showed better efficacy than Src targeting (**Figure 6A**). Comparing the intrinsic caspase 3/7 activity elicited by drug administration revealed much higher apoptosis induced in combinations with lapatinib than afatinib, indicating EGFR/ERBB2 targeting to be a more potent combination with DDR1/BCR-ABL1, as compared to targeting EGFR/ERBB4 (**Figure 6A**).

#### 3.6.2 Combinatorial Efficacy of Nilotinib with Lapatinib

Since nilotinib exhibited a higher inhibitory effect as a single agent and has comparable percent inhibition to other BCR-ABL multi-tyrosine kinase inhibitors when administered in combination with lapatinib, we studied it to see its efficacy in clonal cell proliferation assays. We observed similar cytotoxic effects of nilotinib and bafetinib when combined with lapatinib in the clonal proliferation of HCT116, a COAD line harboring KRAS^G13D^ mutation (**Figure 6B**), and nilotinib in combination was also effective against proliferation of KRAS^WT^ COAD line CaCo2 (**Supplementary Figure 7B**). To confirm the pathway inhibition *via* administration of inhibitors DDR1 7rh and nilotinib in combination with lapatinib, we administered these drugs at sublethal doses on multi-spheroids of HCT116 and performed Western blotting at 48 h posttreatment. Indeed, as predicted by the signaling interactome, co-inhibition of DDR1/BCR-ABL1 signaling with EGFR/ERBB2 decreased the phospho-BCR and phosphor-AKT levels; however, ERK signaling was less affected (**Figure 6C**). This indicates that DDR1 mediates its signaling *via* the BCR-KRAS-PI3K/AKT axis, which became more profoundly inhibited when lapatinib and DDR1 inhibitors were co-administered at doses of their observed synergy. Inhibition of the BCR-ABL1 axis alone using PROTAC inhibitor (GMB475) with lapatinib was used in parallel to compare the inhibition of phospho-AKT downstream of BCR. GMB475 is a degrader of ABL1 protein. Western blotting for degradation of ABL1 and loss of phospho-BCR levels upon administration of GMB475 are included in **Supplementary Figure 7A**.

Nilotinib plus lapatinib administration impacted multi-spheroid viability for both KRAS-mutant COAD lines HCT116 and SW480 (**Figures 6A, D**), and the synergy of these drugs was confirmed in HCT116 multi-spheroids (**Supplementary Figure 5D**). Since nilotinib and lapatinib are brain penetrant, investigating whether their combination can offer better outcomes in GBM tumoroids cultured *in vitro* revealed loss of spheroid viability in GBM line U251, and the two drugs synergized in U251 multi-spheroids (**Figures 6E**, **Supplementary Figure 5D**). Since DDR1 is a common target of all BCR-ABL multi-tyrosine kinase inhibitors (82), to understand whether similar cytotoxicity could be obtained with DDR1 specific inhibitor, we evaluated the effect of DDR1 7rh plus lapatinib on clonal cell proliferation of HCT116 and on spheroid viability of GBM (U251) line (**Supplementary Figure 7B**; **Figure 6E**). We also confirmed the efficacy of inhibiting DDR1/BCR signaling (using nilotinib) in combination with lapatinib on oversized multi-spheroids of COAD line HCT116 and GBM line U251 and found significant loss of spheroid viability (**Supplementary Figure 7C**). Additionally, a positive correlation was observed between the IC_50_ value of lapatinib and nilotinib (Rp value >0.3) in both the Pan-Cancer and COREAD cell line datasets (source: GDSC database), indicating that these signaling cascades are complementary (*Excel sheet 3, sub-sheet S3.3, S3.4*). Moreover, resistance to lapatinib and nilotinib treatment alone was associated with KRAS mutation phenotype in both COREAD and Pan-Cancer (*Excel sheet, sub-sheet 3.5*), emphasizing the value of combinatorial regime against KRAS mutant tumors.

### 3.7 Targeting DDR1/BCR-ABL With EGFR/ERBB2 Against Radioresistance

**Figure 7:**
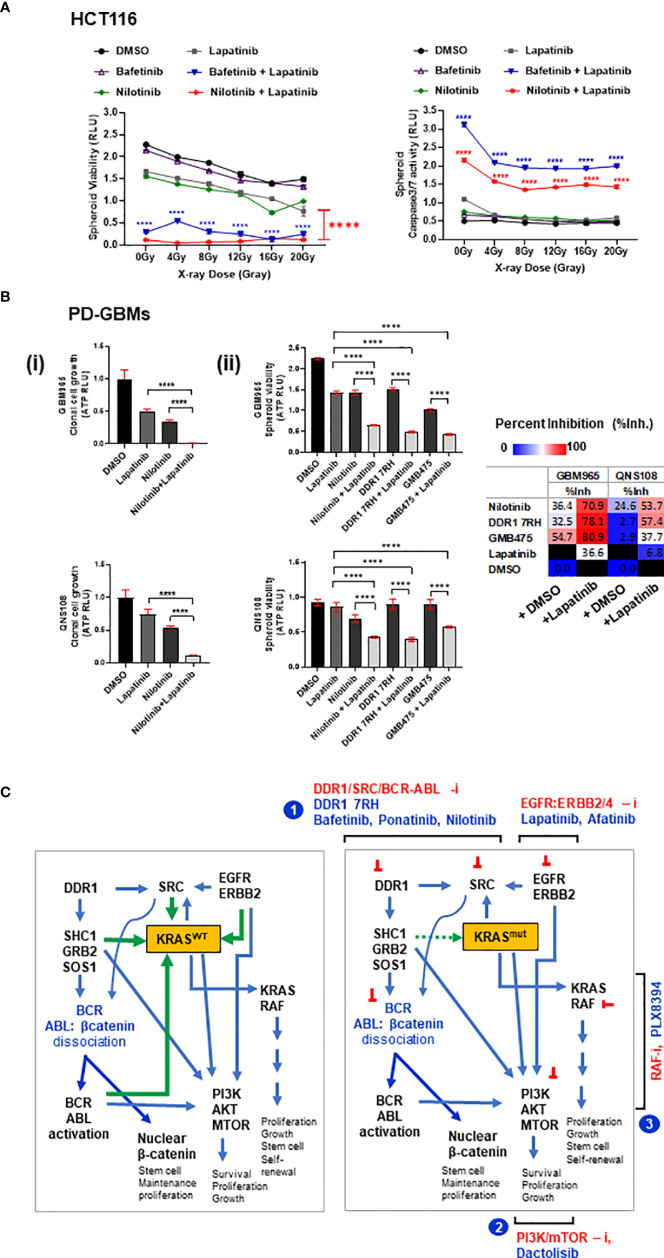
**(A)** HCT116 multi-spheroids that recovered from radiation stress induced by administering IR doses from 0 up to 20 Gy were measured for **(i)** spheroid viability and **(ii) c**aspase 3/7 activity at day 5 post-IR. p-Values for combination treatment of drugs (bafetinib or nilotinib) with lapatinib are marked with asterisks at each of the respective IR doses administered. **(B)** Combinatorial efficacy of nilotinib plus EGFR inhibitor lapatinib on spheroid formation and viability of patient-derived GBM (GBM-PD) lines GBM965 and QNS108. The intrinsic radiosensitivity of these lines is included in the Supplementary Methods. **(C)** Proposed model illustrating the signaling networks involving DDR1, SRC kinases, BCR-ABL-β-catenin, EGFR/ERBB2, KRAS, and PI3K/AKT proteins, and site of action of the leads identified. Illustration (left) shows KRAS^WT^ cross-talks: activated DDR1 relays its effect *via* adaptor proteins SHC1/GRB2/SOS1, causing dissociation of BCR-ABL-β-catenin complex (inactive), thereby activating BCR/ABL proteins. BCR and ABL proteins interact to cause activation of KRAS^WT^ and PIK3/AKT signaling axis. SRC kinase-activated downstream of DDR1 can engage with KRAS in a positive feedback loop. Additionally, EGFR/ERBB2 receptor complex can activate KRAS-dependent MAPK signaling and PIK3/AKT signaling; activated KRAS can also activate PI3K/AKT axis. Green arrows indicate input signals that activate KRAS^WT^, and blue arrows indicate output signals from KRAS to other signaling intermediates. Illustration (right) depicts these signaling interactions with KRAS^mut^ protein, which is nearly constitutively active, thus independent of input signals. Targeting the network of hyperactivated KRAS or KRAS^mut^ comprises the following scheme: 1) combinatorial targeting of DDR1 and BCR-ABL1 by specific inhibitors of DDR1 or DDR1/BCR-ABL by multi-tyrosine kinase inhibitors (bafetinib, ponatinib, and nilotinib) in combination with EGFR inhibitors lapatinib and afatinib. Other potential combinations identified are represented as schemes (2) and (3): involving PI3K/mTOR inhibition by dactolisib, in combination with EGFR inhibitors lapatinib and afatinib or KRAS/RAF axis inhibition by PLX8394 in combination with EGFR inhibitors lapatinib and afatinib. These combinatorial schemes can be harnessed to overcome the treatment resistance observed in WNT-activated (stem-like) KRAS mutant tumors. Statistical significance is indicated as ****p < 0.0001.

The cytotoxic potential of the combination of drugs bafetinib and nilotinib with or without lapatinib was evaluated by administering the drugs on irradiated multi-spheroids from HCT116 and measuring spheroid viability and Caspase 3/7 activity (apoptosis) at day 5 post-IR. Significant reduction in spheroid viability and increase in apoptosis were observed in combination treatments (bafetinib or nilotinib plus lapatinib) as compared to control groups at all IR doses administered (**Figure 7A**). The combinatorial effect of nilotinib plus lapatinib was further confirmed on patient-derived GBM (PD-GBM) lines GBM965 and QNS108 (intrinsically radioresistant), and both the lines showed pronounced growth reduction in combination treatment [**Figure 7B**(**i**)]. To evaluate the effect of targeting the DDR1/BCR-ABL1 axis in combination with lapatinib on multi-spheroid viability, the PD-GBMs were cultured for 6 days, drugs were administered, and viability was measured at day 2 posttreatment. A significant reduction in viability was observed for all three combinations (nilotinib, DDR1 7rh, or GMB475) with lapatinib. GBM965 was identified as a fast proliferative line as compared to QNS108 (data not shown). Relatively high percent inhibition values were obtained for GBM965 when administered with any of the three drugs as a single agent or in combination, which could be due to cell line-specific intrinsic genomic or proteomic profiles that account for its survival and growth. To evaluate this, we utilized the gene expression dataset (GSE144610) available for GBM965 (**Supplementary Figure 8**). We observed significantly higher reads (counts-normalized) for genes EGFR and ERBB2 as compared to ERBB3/4 and high expression of BCR, ABL1, KRAS, BRAF, and CTNNB1 (β-catenin). Positive regulators of KRAS signaling (PTPN2 and PTPN11) had higher read counts than the NF1 gene (a negative regulator of KRAS). Moreover, PI3K/MTOR proteins had higher read counts as opposed to their negative regulator protein PTEN. Being a proliferative line, and with high intrinsic radioresistance (**Supplementary Method 1**), it is likely to engage activated WNT and KRAS cross-talk, which led to its observed sensitivity to combinatorial targeting of DDR1/BCR-ABL and EGFR/ERBB2.

## 4 Discussion

WNT/β-catenin and KRAS signaling co-operate in cancer progression and are the leading cause of establishment of treatment resistance and poor patient outcomes (17–19, 41, 42). WNT pathway is regulated at the receptor level by R-spondin/Lgr5 and intracellularly by the β-catenin destruction complex comprising the APC protein (83–85). KRAS is the main downstream effector of EGFR-mediated MAPK signaling and is one of the most prevalent oncogenic drivers in COAD. KRAS activity is regulated at two levels: i) conversion of KRAS GDP-bound form (inactive state) to KRAS GTP-bound form (active) and ii) dephosphorylation of tyrosine residues by phosphatase SHP2 (PTPN11) (55–60). The gain-of-function mutations at G12 or G13 in KRAS render the protein independent of the GTP-GDP on–off switch, making it nearly constitutively active (5, 6). While drugs to directly target WNT and mutant KRAS are in clinical trials, their moderate efficacy and potential cytotoxicity have drawn attention to identifying their targetable regulators and signaling cross-talks (55–68). We approached this challenge through an unbiased drug screen, targeting receptors and signaling intermediates that relate to WNT and KRAS and their downstream cellular processes like survival, proliferation, CSC reactivation, adhesion, migration, and epigenetic modulation with the aim to identify novel combinatorial leads that could synergize with small-molecule inhibitors of wild-type EGFR to combat treatment-resistant WNT-activated KRAS-driven tumors. In our study, we utilized the potential of three-dimensional (3D) multi-spheroid assays since increasing evidence suggests 3D tumoroids recapitulate a more near-physiological state retaining properties of its cell of origin and can generate ECM for compaction and signaling, thus serving as better model systems for evaluation of treatment resistance and response (86–89).

In our drug screen, we identified 5 potential combinatorial leads (PLX8394, bafetinib, ponatinib, VU6015929, and DDR1 7rh) with EGFR inhibitors (lapatinib and afatinib); bafetinib and ponatinib showed the best response in all COAD lines, independent of KRAS mutation type. PLX8394, a dimerization inhibitor of BRAF investigated frequently against tumors harboring mutant-BRAF (51) showed a better combinatorial response with lapatinib since RAF proteins are prime mediators of the KRAS-RAF-MEK-ERK signaling axis. The overlapping targets among the remaining 4 drugs included the following: i) discoidin domain receptor 1 (DDR1) protein, a receptor tyrosine kinase involved in cell–cell and cell–matrix adhesion and upregulated in several cancers (DDR1 was identified as a common target among all 4 drugs, namely, bafetinib, ponatinib, DDR1 7rh, and VU6015929); ii) Src kinases (Lyn/Src); iii) breakpoint cluster region (BCR) protein, an activator of GEF (Guanosine nucleotide exchange factor), involved in oncogenic transformations; and iv) ABL1 kinase, involved in growth, survival, and cytoskeletal remodeling. BCR-ABL fusion is the key genomic aberration in chronic myelogenous leukemia (CML). Independent of this genomic alteration, BCR and ABL proteins co-operate to activate KRAS signaling (90, 91). Cross-talk between WNT/β-catenin and BCR-ABL has also been reported in CML, which contributes to treatment resistance (92). A recent study showed DDR1 as a therapeutic target in colon cancer, activating BCR signaling downstream (68). SRC kinases are known to associate with receptor tyrosine kinases (including EGFR and DDR1) and regulate BCR activation (93–96). Together, this explains an active signaling network involving KRAS, SRC, DDR1, BCR, and ABL proteins.

We observed potential synergy between these 5 combinatorial leads with EGFR/ERBB2 small-molecule inhibitor, lapatinib. This is because cetuximab-resistant tumors are found to have mutational hotspots in EGFR/ERBB2, and ERBB2 amplifications contribute to the pathophysiology of advanced CRCs (37–40). Moreover, a recent study in breast cancer reported DDR1 to be an interacting partner of ERBB2, and upregulation of DDR1 is one of the compensatory survival mechanisms in lapatinib-resistant tumors (97, 98). Our genomic analysis revealed ERBB2, DDR1, BCR, and LYN (Src kinase) genes for WNT regulation (LGR5) were upregulated in COAD and DDR1 to positively correlate with BCR, EGFR, and ERBB2 in Pan-Cancer, all of which can emphasize the possible synergy of EGFR/ERBB2 inhibition with inhibitors of DDR1-BCR-ABL signaling.

GBM tumors are highly resistant to current therapies, and increasing evidence shows the role of activated WNT/β-catenin, PI3K/mTOR, and RAS signaling in GBM progression and recurrence (99–102). EGFR amplification and NF1 inactivation mutations are observed in GBMs in a subtype-dependent manner (72, 73) and lead to activation of KRAS (103–105). Moreover, activated KRAS is observed to contribute to tumor stemness and invasiveness post-radiation therapy, making it a potential therapeutic target in recurring GBMs (106). DDR1 was identified as a neurotrophic receptor tyrosine kinase (NTRK4) highly upregulated in cancers and is documented to have therapeutic value in GBM (107–111). We observed DDR1 expression to be highly elevated in COAD lines (HCT116 and DLD1) and patient-derived GBM line GBM965. We therefore conducted a comparative investigation of targeting DDR1 in combination with EGFR inhibitor (lapatinib), utilizing an additional tumor model of GBM and including the brain penetrable DDR1/BCR-ABL inhibitor nilotinib to evaluate its combinatorial efficacy. Our data showed significant cytotoxicity by nilotinib plus lapatinib treatment in multi-spheroids of COAD and GBM lines, in primary patient-derived GBMs, and in HCT116 multi-spheroids that recovered from radiation stress induced by 20 Gy-IR, indicating DDR1/BCR-ABL axis with EGFR-ERBB2 targeting as treatment opportunity against chemoradioresistant cancers.

At the genomic level, we observed transcriptional expression of genes BRAF, PI3K, MTOR, BCR, and APC to positively correlate with DDR1 in patient datasets of COAD and GBM. Additionally, the expression of genes EGFR, ERBB2, ERBB4, BCR, SOX9, VANGL2, and CDH1 correlated with DDR1 in Pan-Cancer. BRAF, PI3K, and MTOR contribute to signaling downstream of KRAS, and BCR activates KRAS signaling in association with SRC and ABL kinases. SOX (SRY-Box transcription Factor) proteins are upregulated in several different tumors (112), and increasing evidence has shown its potential in the induction of stemness and treatment resistance in CRC (113–116). The transmembrane protein Vang-like 2 (VANGL2) is required for planar cell polarity and plays a role in cell adhesion, directed cell migration, and metastasis (117–119). We also observed a significant positive correlation between the protein levels of DDR1 and beta-catenin in Pan-Cancer. The positive association between the expression of DDR1 with genes SOX9, CTNNB1, or VANGL2 emphasizes its role in stemness and tumor aggressivity. Positive correlation in expression of genes DDR1, BCR, EGFR, and ERBB2 further points to the wide applicability of targeting the DDR1/BCR axis with EGFR/ERBB2 in precision medicine. Investigating the protein interactome of DDR1 with adaptor proteins (SHC1, GRB2, and SOS1) to understand how these interconnect DDR1, BCR, ABL, KRAS, and PI3K signaling revealed involvement of BCR-ABL and KRAS-PI3K-AKT axis downstream of DDR1, which was confirmed by Western blotting. Taken together, our work shows that targeting DDR1, BCR-ABL, or DDR1/BCR-ABL with EGFR-ERBB2 could offer a potential therapeutic strategy against WNT-activated (stem-like) and KRAS-mutant COAD, with translatable applications in stem-like refractory cancers such as GBMs.

## 5 Conclusion

We identified the signaling pathways that could be targeted in combination with small-molecule inhibitors of wild-type EGFR to overcome the stem-like KRAS mutant phenotype in tumoroids of COAD. These involved three combinatorial strategies: i) targeting DDR1, BCR-ABL, and EGFR-ERBB2/4; ii) targeting Pan-RAF/BRAF with EGFR-ERBB2/4; and iii) targeting PI3K, MTOR, and EGFR-ERBB2/4 (schemes illustrated in **Figure 7C**). Among these three approaches, we emphasized targeting of DDR1/BCR axis with EGFR-ERBB2 since DDR1 was the most prevalent target among all the combinatorial leads we identified. We found inhibition of DDR1/BCR signaling with EGFR/ERBB2 to be effective independent of KRAS mutation type and status in COAD and additional primary tumor model of GBM. Independent groups have reported DDR1 as a potential therapeutic candidate in KRAS-driven tumors (66, 66, 120), and combinatorial targeting of DDR1 has also been shown to be efficacious in KRAS-mutant tumors, lung adenocarcinoma (67), and recently pancreatic adenocarcinoma (121). We show for the first time a comprehensive investigation on identifying targeted therapies against cetuximab-resistant stem-like KRAS-driven tumors by exploring the inhibitors against a wide spectrum of signaling pathways and revealing the complementation of DDR1/BCR-ABL axis with EGFR-ERBB2, which could be harnessed to combat chemoradioresistance mediated by high β-catenin and activated KRAS. Underpinning the mechanisms of how DDR1/BCR-ABL targeting works against KRAS-mutant tumors requires further investigation may involve limiting the stimuli for cell adhesion, survival, anti-apoptotic, and proliferation factors, all of which may co-operate with mutant KRAS to exhibit tumor aggressivity. A recent study showed targeting DDR1 as a therapeutic strategy against resistance to BRAF and MEK inhibitors (81), further confirming the significance of our findings, which suggest an unprecedented role of the interactome of DDR1, BCR, and ABL1 proteins with EGFR-ERBB2-4 signaling in tumor progression. The only FDA-approved drugs currently available for DDR1 and BCR/ABL proteins are the BCR-ABL family of multi-tyrosine kinase inhibitors; however, DDR1- and BCR-ABL-specific compounds are constantly being developed (82, 122), and a combination of DDR1-, BCR-ABL1-, and EGFR/ERBB2-4-specific drugs could co-operate, synergize, and open a new paradigm for future cancer therapeutics.

## 6 Future Perspectives

KRAS is one of the oldest and most potent oncogenes identified, along with MYC and ABL1 (123, 124). Its role as a signaling effector downstream of MAPK signaling and in cell cycle progression has been well established across species (125, 126). While efforts have been made in the direct targeting of KRAS mutant isoforms, the necessity to identify the granularities of KRAS signaling interactome has become imperious, and efforts are being made to decipher new combinatorial targeted therapies to combat the highly resilient phenotype of RAS pathophysiology.

Our study demonstrates a strong synergy between drugs targeting DDR1/BCR signaling and EGFR/ERBB2, and we show here a consistently favorable outcome with combination therapy engaging proteins DDR1, BCR, ABL1, and EGFR/ERBB2 in multiple lines of COAD independent of their KRAS mutation type specificity, as well as in a second tumor model of recurrent GBM. However, there are a few limitations of this work: i) our findings are based on *in vitro* 3D tumoroid models; therefore, it is necessary to evaluate their pharmacokinetics and response *in vivo*. ii) The study limits itself to evaluating synergy between two drugs in every combination; however, identifying the interactions between multiple drugs in a combination treatment regime and extrapolating these studies to a large cohort of patient samples require high-throughput automated equipment, and accurately predicting the outcome of combination therapy in a clinical setting is a multidisciplinary effort (127–129). Despite these challenges, combination therapies offer great advantages over monotherapy, and their applications in cancer treatment continue to grow (130–134). This study points to the significance of complementary cascades and pathway shunts that play a role in augmenting treatment resistance, highlighting the significance of combination therapies in recurrent tumors. DDR1 is a receptor for ECM proteins, collagens, which became overexpressed as tumors progress, and plays a central role in angiogenesis, tumor cell migration, and invasion (135–137). Stromal expression of DDR1 has also been shown to promote the growth of tumors harboring WNT-activated phenotype (138), and collagens have been widely documented as prognostic markers in colon cancer progression to metastasis (139–146). ECM deposition poses a challenge to immune cell infiltration in tumors and impairs treatment efficacy, and ECM signaling has emerged as a potential target for cancer therapy (147–151). Moreover, the effects of conventional radiotherapy (RT) on tumor stroma and ECM composition with exacerbating tumor-promoting phenotype in a KRAS-dependent manner in GBM has been documented and reviewed (106, 152). Intense research is therefore ongoing in the field of high-precision RT to improve patient outcomes (153–157). If upregulation of signaling pathways engaging RTKs like EGFR, ERBB2, and DDR1 and proteins like BCR and ABL1 can collectively contribute to aggressivity and poor response in KRAS-driven tumors, there may be an opportunity to harness insights from this study to further their combinatorial potential with other targeted inhibitors and advanced treatment modalities, like immunotherapy and precision radiation therapy to enhance efficacy in a clinical setting and to widen their scope in personalized medicine.

## Data Availability Statement

Publicly available datasets were analyzed in this study. The datasets used in this study can be found in online repositories TCGA, GTEx, GEO, and GDSC and genomics portals GEPIA and cBioPortal. Protein interactome data are available at BioGRID, STRING, and Pathway Commons. The names of the repositories, as applicable, can be found in the article/Excel sheets—**Supplementary Material**.

## Author Contributions

KG performed study proposition, design, resource organization, experimentation, data curation, data analysis, co-investigation, and writing—reviewing and editing. JJ investigated the study with KG. VF performed radio-sensitization evaluation on GBM-PD lines, AQH Lab. YM and PS helped with laboratory resource organization. SK: investigation, funding, and support. The figures were by KG and VF. Illustrations were by KG. All authors contributed to the review/editing and conception of the final manuscript.

## Funding

This study was funded in part by the National Institutes of Health grants U01CA216468 and R01CA257241 (SK). Additionally, the work was supported by the RACER grant (KG, JJ, and SK).

## Conflict of Interest

The authors declare that the research was conducted in the absence of any commercial or financial relationships that could be construed as a potential conflict of interest.

## Publisher’s Note

All claims expressed in this article are solely those of the authors and do not necessarily represent those of their affiliated organizations, or those of the publisher, the editors and the reviewers. Any product that may be evaluated in this article, or claim that may be made by its manufacturer, is not guaranteed or endorsed by the publisher.

## Abbreviations

CRC, colorectal cancer; COAD/COREAD, colorectal adenocarcinoma; GBM, glioblastoma multiforme; GBM-PD, GBM patient-derived cell lines; WNT, wingless-type MMTV integration site family; APC, adenomatous polyposis coli; CTNNA1, catenin-Alpha1 gene; CTNNB1, catenin-Beta1 gene; EGF, epidermal growth factor; FGF2, fibroblast growth factor 2; EGFR, epidermal growth factor receptor; ERBB2/3/4, V-Erb-B2 erythroblastic leukemia viral oncogene homolog 2/3/4; MAPK, mitogen-activated protein kinase; KRAS, Kirsten Rat Sarcoma Viral Oncogene Homolog; NRAS, neuroblastoma RAS Viral (V-Ras) oncogene homolog; WT-KRAS/KRAS^WT^, wild-type KRAS; KRAS^mut^, mutant KRAS; PI3K, phosphatidylinositol-4,5-bisphosphate 3-kinase; PIK3CA, phosphatidylinositol-4,5-bisphosphate 3-kinase catalytic subunit-α; AKT, V-Akt (Ak strain transforming) murine thymoma viral oncogene; MTOR, mammalian target of rapamycin kinase; RAF, V-Raf murine leukemia viral oncogene-like protein; BRAF, V-Raf murine sarcoma viral oncogene homolog B1; TGFβ, transforming growth factor-beta; SRC, V-Src avian sarcoma (Schmidy-Ruppin A-2) viral oncogene homolog; LYN, Lck/Yes related novel protein tyrosine kinase; CDH1, cadherin-1; DDR1, discoidin domain receptor 1; NTRK, neurotrophic receptor tyrosine kinase; CAK, cell adhesion kinase; RTK, receptor tyrosine kinase; MTK, multi-tyrosine kinase; BCR, breakpoint cluster region; ABL/ABL1, Abelson Murine Leukemia Viral Oncogene Homolog 1; LGR5, leucine-rich repeat containing G-protein-coupled receptor-5; SHC1, SHC (Src-homology 2 domain containing) transforming protein 1; GRB2, growth factor receptor bound protein 2; SOS1, Son of Sevenless Homolog 1; SOX, SRY-Box transcription Factor; VANGL2, Van Gogh-Like Planar cell polarity protein 2; PTPN11, protein tyrosine phosphatase non-receptor type 11; ECM, extracellular matrix; NF1, neurofibromin-1; PTEN, phosphatase and tensin homolog deleted on chromosome 10; ATP, adenosine nucleotide triphosphate; GTP, guanosine nucleotide triphosphate; GDP, guanosine nucleotide diphosphate; RT, radiation therapy; CRT, chemoradiation therapy; IR, irradiation; ROS, reactive oxygen species; Gy, Gray; mut (superscript), mutant; WT (Superscript), wild-type; i (suffix), Inhibitor; EGFRi, EGFR inhibitor; N2EF, N2N21max, N2+EGF+FGF, N2+N21max; TCGA, The Cancer Genome Atlas; GTEx, Genotype Tissue Expression; GEPIA, gene expression profiling interactive analysis; GDSC, Genomics of Drug Sensitivity in Cancer; IC_50_, inhibitory dose 50; Rs/Rp, Spearman’s/Pearson’s correlation coefficients.

## Glossary

CRC: colorectal cancer
COAD/COREAD: colorectal adenocarcinoma
GBM: glioblastoma multiforme
GBM-PD: GBM patient-derived cell lines
WNT: wingless-type MMTV integration site family
APC: adenomatous polyposis coli
CTNNA1: catenin-Alpha1 gene
CTNNB1: catenin-Beta1 gene
EGF: epidermal growth factor
FGF2: fibroblast growth factor 2
EGFR: epidermal growth factor receptor
ERBB2/3/4: V-Erb-B2 erythroblastic leukemia viral oncogene homolog 2/3/4
MAPK: mitogen-activated protein kinase
KRAS: Kirsten Rat Sarcoma Viral Oncogene Homolog
NRAS: neuroblastoma RAS Viral (V-Ras) oncogene homolog
WT-KRAS: wild-type KRAS
KRAS^mut^: mutant KRAS
PI3K: phosphatidylinositol-4,5-bisphosphate 3-kinase
PIK3CA: phosphatidylinositol-4,5-bisphosphate 3-kinase catalytic subunit-α
AKT: V-Akt (Ak strain transforming) murine thymoma viral oncogene
MTOR: mammalian target of rapamycin kinase
RAF: V-Raf murine leukemia viral oncogene-like protein
BRAF: V-Raf murine sarcoma viral oncogene homolog B1
TGFβ: transforming growth factor-beta
SRC: V-Src avian sarcoma (Schmidy-Ruppin A-2) viral oncogene homolog
LYN: Lck/Yes related novel protein tyrosine kinase
CDH1: cadherin-1
DDR1: discoidin domain receptor 1
NTRK: neurotrophic receptor tyrosine kinase
CAK: cell adhesion kinase
RTK: receptor tyrosine kinase
MTK: multi-tyrosine kinase
BCR: breakpoint cluster region
ABL/ABL1: Abelson Murine Leukemia Viral Oncogene Homolog 1
LGR5: leucine-rich repeat containing G-protein-coupled receptor-5
SHC1: SHC (Src-homology 2 domain containing) transforming protein 1
GRB2: growth factor receptor bound protein 2
SOS1: Son of Sevenless Homolog 1
SOX: SRY-Box transcription Factor
VANGL2: Van Gogh-Like Planar cell polarity protein 2
PTPN11: protein tyrosine phosphatase non-receptor type 11
ECM: extracellular matrix
NF1: neurofibromin-1
PTEN: phosphatase and tensin homolog deleted on chromosome 10
ATP: adenosine nucleotide triphosphate
GTP: guanosine nucleotide triphosphate
GDP: guanosine nucleotide diphosphate
RT: radiation therapy
CRT: chemoradiation therapy
IR: irradiation
ROS: reactive oxygen species
Gy: Gray
mut (superscript): mutant
WT (Superscript): wild-type
i (suffix): Inhibitor
EGFRi: EGFR inhibitor
N2EF: N2N21max, N2+EGF+FGF, N2+N21max
TCGA: The Cancer Genome Atlas
GTEx: Genotype Tissue Expression
GEPIA: gene expression profiling interactive analysis
GDSC: Genomics of Drug Sensitivity in Cancer
IC_50_: inhibitory dose 50
Rs/Rp: Spearman’s/Pearson’s correlation coefficients
