# Supplementary material for: Identification of Synergistic Drug Combinations to Target KRAS-Driven Chemoradioresistant Cancers Utilizing Tumoroid Models of Colorectal Adenocarcinoma and Recurrent Glioblastoma
Source: Front Oncol. 2022 May 18;12:840241. doi: 10.3389/fonc.2022.840241 (PMC9158132; doi:10.3389/fonc.2022.840241)
Supplement: Supplementary file 1 [file DataSheet_1.pdf]

SUPPLEMENTARY METHODS

1. Radiosensitivity curves for QNS965 and QNS108

Intrinsic radiosensitivity of GBM lines

Radiosensitivity curves for radioresistant GBM lines were build as described previously.[1] Briefly, GBM cells seeded on sextuplicates on ultra-low attachment 96 well plates received a single dose of 0, 2, 4, or 6 Gy (275.1 cGy/min) with an X-rad 1600 (Precision X-Ray). Plating efficiency was determined after 3 weeks considering the number of spheres with more that 100 µm of diameter. Surviving fractions at each dose were then fitted to the linear quadratic model with R studio[2] using the CFAssay R package.[3] Surviving fractions at 2 Gy (SF2) were used to determine sensitivity to radiation, GBM cell lines with SF2 > 40% were considered radioresistant.

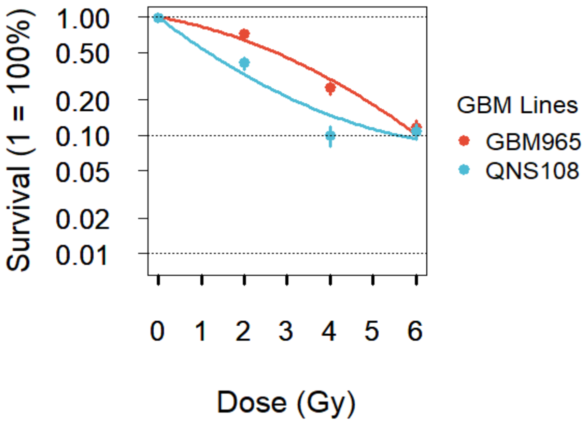

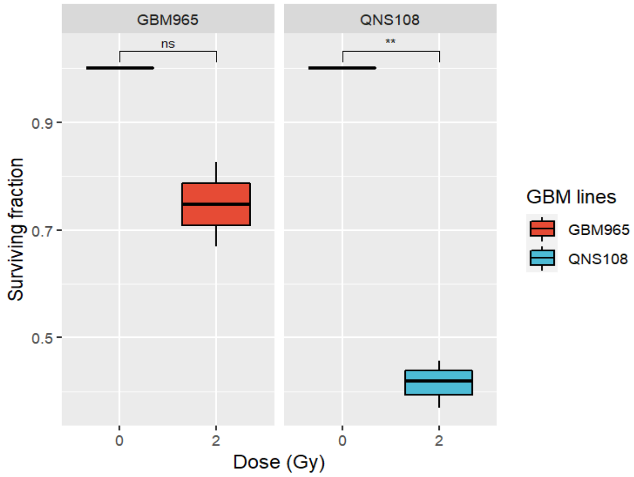

References:

1. Gómez-Millán, J. et al. The importance of bystander effects in radiation therapy in melanoma skin-cancer cells and umbilical-cord stromal stem cells. Radiotherapy and oncology: journal of the European Society for Therapeutic Radiology and Oncology 102, 450–458 (2012).
2. RStudio Team. RStudio: Integrated development environment for r. (RStudio, PBC., 2020).
3. Braselmann, H. CFAssay: Statistical analysis for the colony formation assay. (2020).
