# Supplementary material for: Identification of Synergistic Drug Combinations to Target KRAS-Driven Chemoradioresistant Cancers Utilizing Tumoroid Models of Colorectal Adenocarcinoma and Recurrent Glioblastoma
Source: Front Oncol. 2022 May 18;12:840241. doi: 10.3389/fonc.2022.840241 (PMC9158132; doi:10.3389/fonc.2022.840241)
Supplement: Supplementary file 2 [file DataSheet_2.pdf]

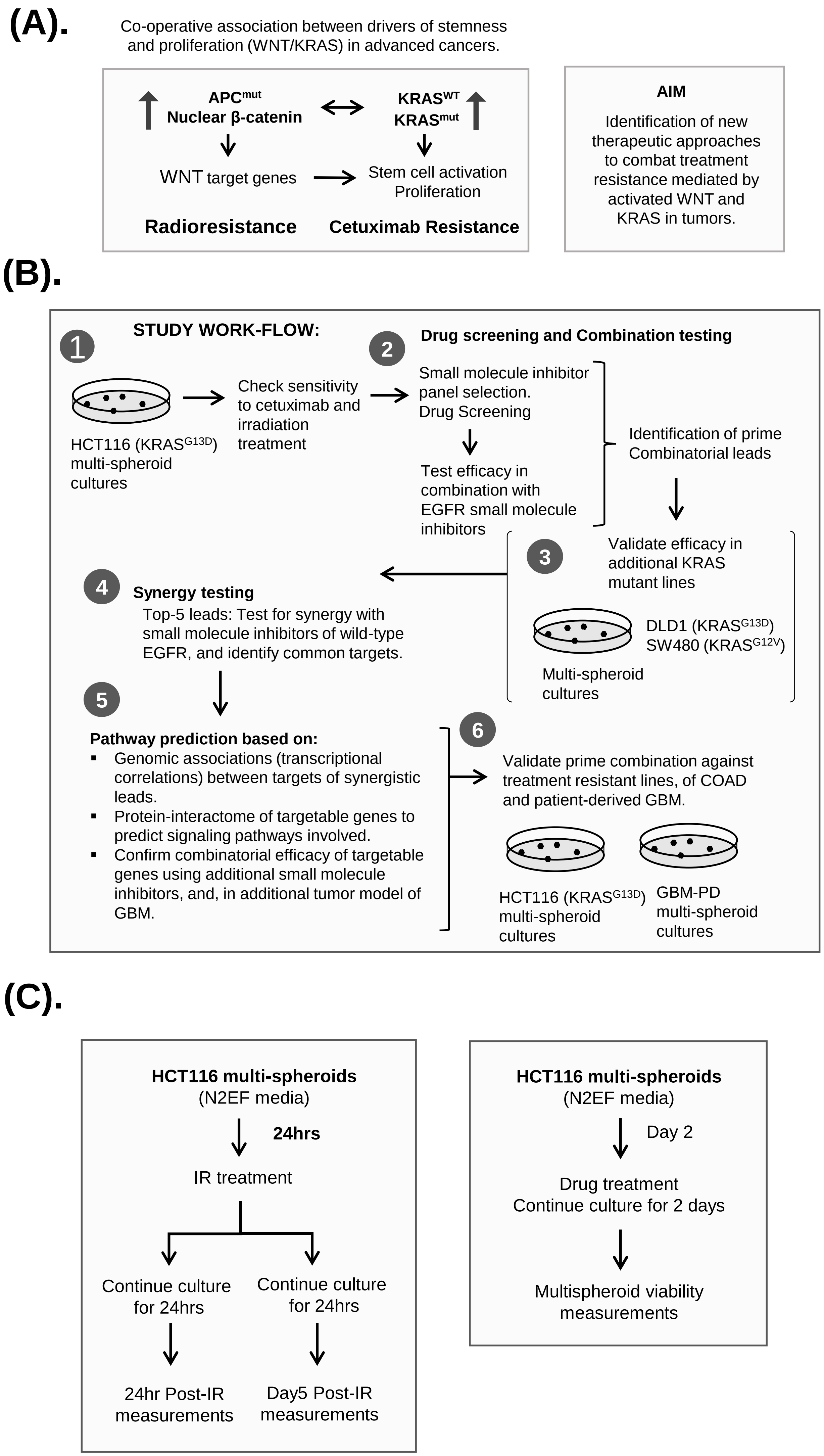

Supplementary Figure 1

Supplementary figure 1: (A). Illustration (Literature-based) to represent that, mutant (inactive) APC protein and high-nuclear β-catenin co-operate bidirectionally with upregulated KRAS signaling. Together, WNT effector activation, and activated KRAS protein mediate radioresistance and cetuximab resistance observed in advanced refractory tumors. To the right is study aim stated. **(B)**. Illustration to depict the study work-flow**. (C). (i)** Experimental scheme to investigate internal resistance to radiation in HCT116 multi-spheroids. **(ii)** Scheme utilized to perform drug treatments and multi-spheroid viability assays in HCT116.

Table 1: Culture conditions used for multi-spheroid growth.

Tumor Type	Cell Lines	Media		Media supplements				
			FBS	N2	N21max	EGF	FGF2	ITSx
COAD	HCT116	DMEM F12	-	+	-	+	+	-
		DMEM F12	-	+	+	-	-	-
COAD	DLD1	RPMI	-	+	+	-	-	-
COAD	SW480	DMEM F12	-	+	+	-	-	-
GBM	U251	DMEM F12	-	+	-	+	+	+
GBM-PD	GBM965	DMEM F12	-	+	+	+	+	+
GBM-PD	QNS108	DMEM F12	-	+	+	+	+	+

(A). **Floating culture of HCT116 and DLD1 in Ultra low attachment 6 well plate.**

Images (4x) in Bright field (BF) at day10 in culture.

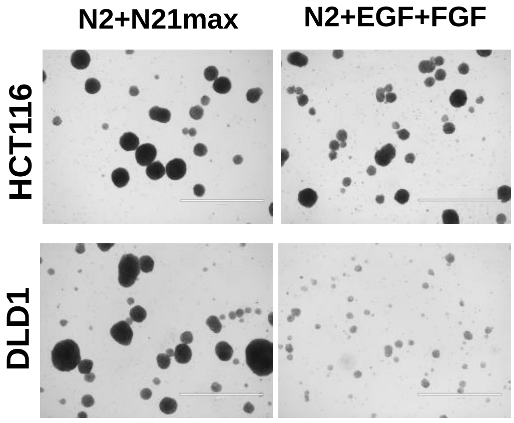

(B). **HCT116 and DLD1: cultured in standard 96 well plate.**

Images (4x) and (10x) in Bright field at day 3 in culture.

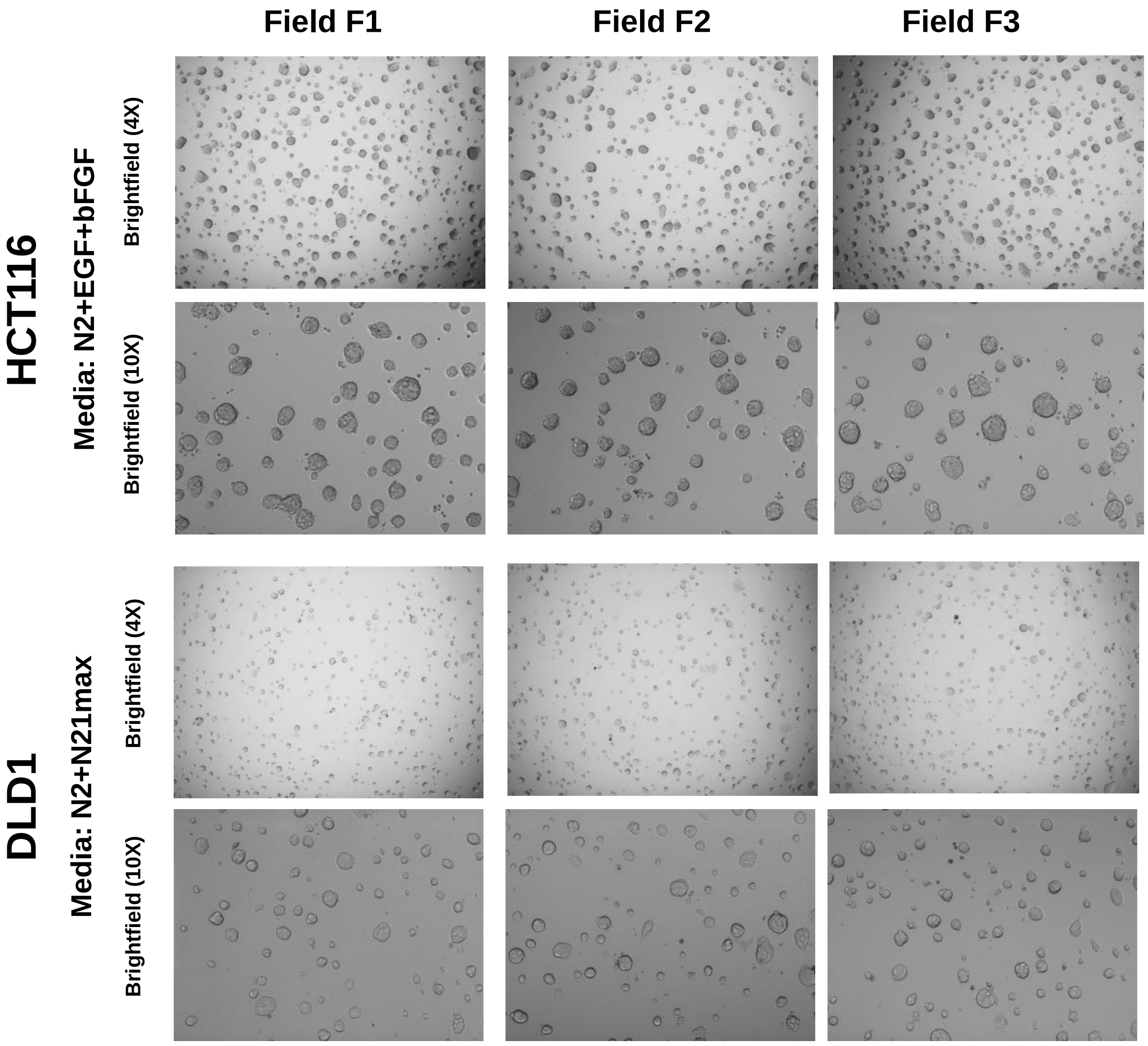

Supplementary Figure 2

(C). Spheroid size and growth evaluation:

(i) Images (4x) in Bright field (BF) at day 7 (1week) in culture.

HCT116 — **DLD1**

N2+EGF+FGF | **N2+N21max** | **N2+N21max**

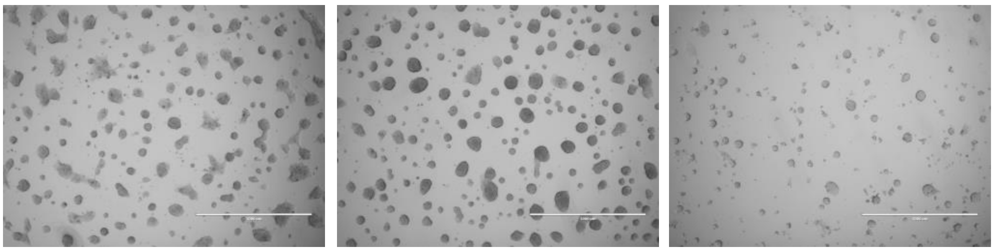

HCT116 — **DLD1**

N2+EGF+FGF | **N2+N21max** | **N2+N21max**

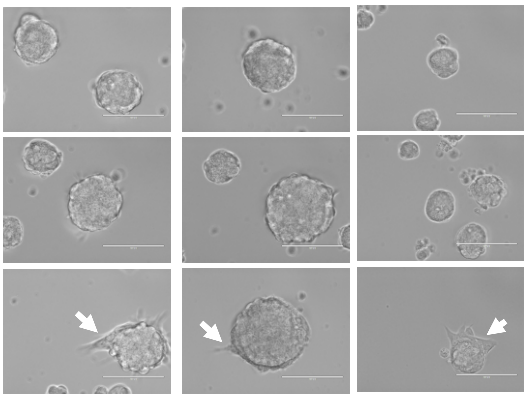

Properties	HCT116		DLD1
Media of growth	N2+EGF +FGF	N2+ N21max	N2+ N21max
Spheroid diameter	40-70µm	50-100µm	20-50µm
Loss of spherical shape at diameter	≥90µm	≥110µm	≥60µm

(ii)

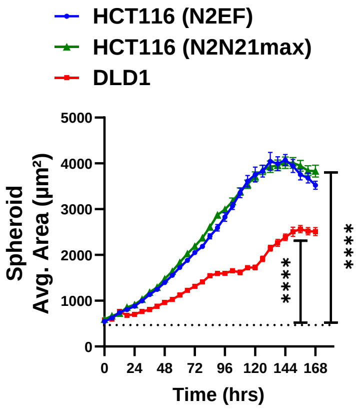

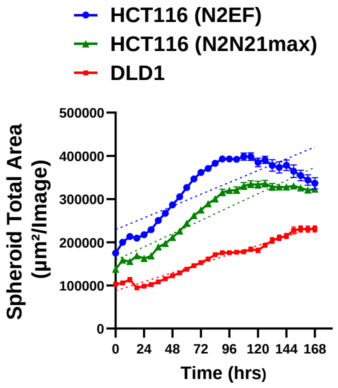

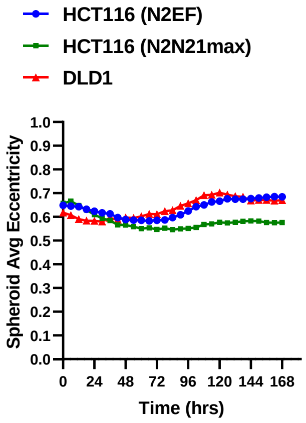

Slope of the curve for Spheroid Total Area		
HCT116 (N2EF)	HCT116 (N2N21max)	DLD1 (N2N21max)
Slope = 1140 , P < 0.0001	Slope = 1282, P < 0.0001	Slope = 876, P < 0.0001

(iii) **SW480 multi-spheroid growth**

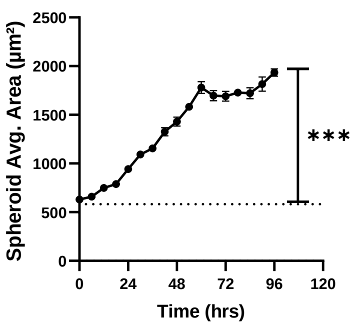

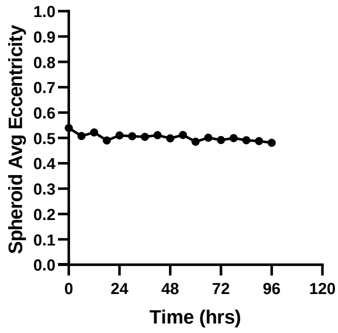

(D). **Evaluation of intrinsic resistance to cetuximab and single-dose IR treatment in multi-spheroids of HCT116.**

(i) HCT116 multi-spheroids cultured in media: N2EFmax.

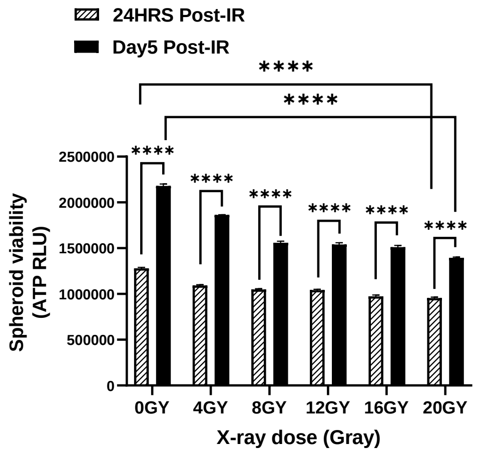

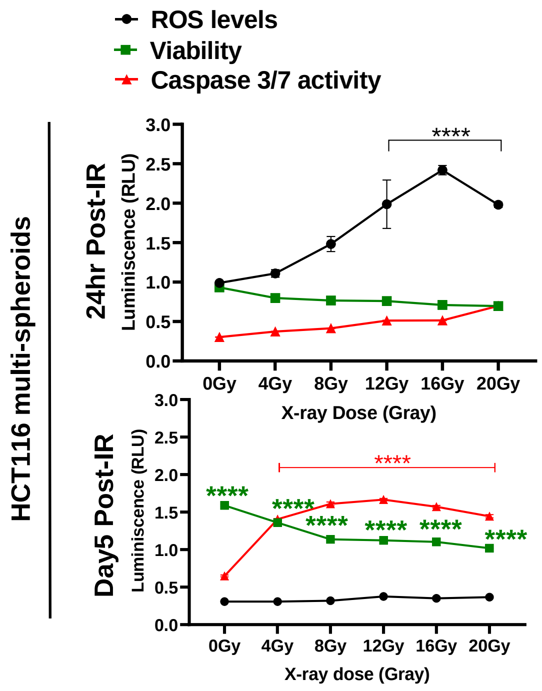

(ii) HCT116 multi-spheroids cultured in media: N2N21max.

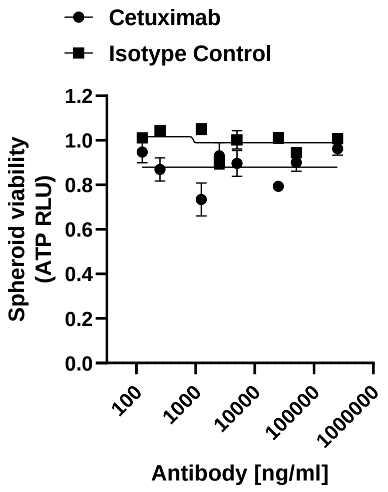

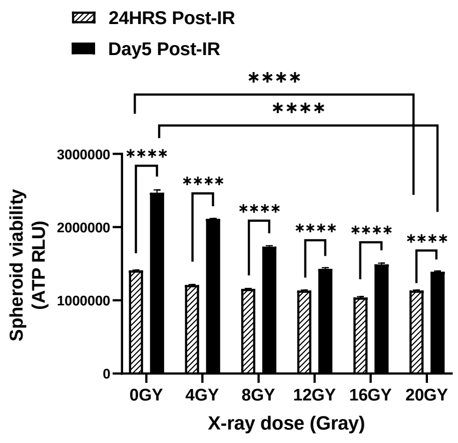

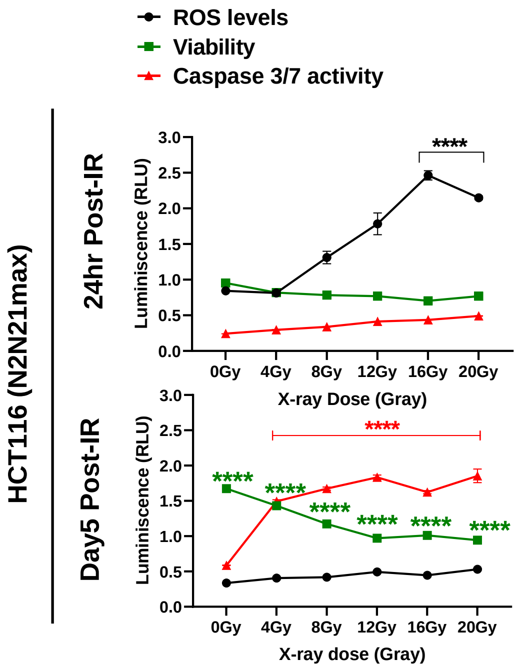

Supplementary Figure 2: Table-1, Culture media conditions used for obtaining spheroids from respective cell lines. **(A).** Representative bright field images (4x) showing spheroids obtained from floating cultures of HCT116 and DLD1 in media conditions indicated. **(B).** Three independent Bright field images (4x, and 10x) of multi-spheroids obtained from HCT116 and DLD1 under indicated media conditions, at day3 in culture. **(C).** Spheroid size and growth evaluation. (i) Bright field (BF) images at 4x, and 40x, for HCT116 cultured in N2EF vs. N2N21max media, and DLD1 in N2N21max media. Approximately 15 independent images under each condition were taken using EVOS FL microscope, and the spheroid diameter was averaged. (ii) Comparing the Spheroid growth parameters, spheroid Average Area (μm^2) and, spheroid total area (μm^2/image) and spheroid average eccentricity for HCT116 and DLD1 multi-spheroids cultured in 96 well plate, under media conditions indicated, and over a period of 168hrs (day7). (iii) Graph (left): Increase in Spheroid average area for SW480 cell line over a period of 5 days. Graph (right), Spheroid average eccentricity for SW480**. (D). (i)** Evaluation of intrinsic resistant to radiation in HCT116 multi-spheroids cultured in N2EF media. Bar graphs indicate that despite notable reduction in growth of spheroids between 0Gy to 20Gy, significant rise in spheroid viability is observed by Day 5post-IR as opposed to spheroids at 24hr-post IR, at each of the radiation doses administered. ROS levels, spheroid viability and Caspase 3/7 activity measured 24hrs post-IR, and, at Day5 post-IR and normalized data for each of the parameters is represented at the right. Significance in change of spheroid viability between 24hr-post IR and Day5 post-IR at respective IR-dose treatment is indicated (****, p-value < 0.0001). (ii) Spheroid viability in response to Cetuximab (anti-EGFR) treatment on HCT116 multi-spheroids cultured in N2N21max media. To the bottom, is **e**ffect of irradiation on growth and survival of HCT116 spheroids (cultured in N2N21max).

(A). Spheroid viability dose-response curves for COAD cell lines.

(i) HCT116 (cultured in media N2EF)

MAPK (KRAS) - i

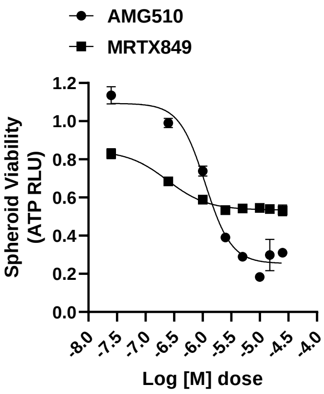

MAPK (RAF) - i

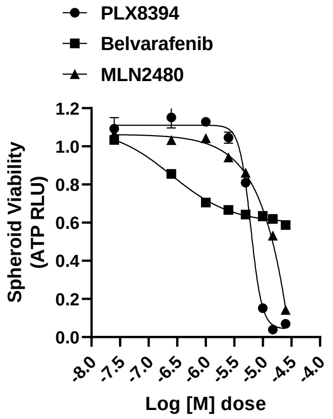

PI3K, PI3K/mTOR - i

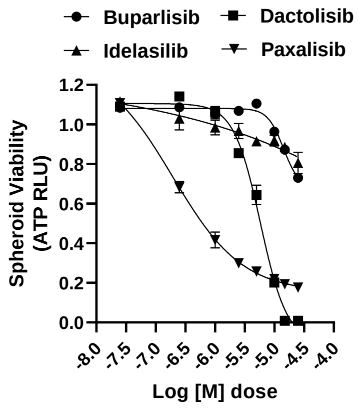

Multi-tyrosine Kinase - i

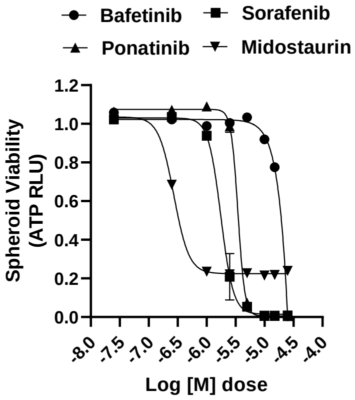

Neurotrophic receptor Tyrosine kinase (NTRK)- i

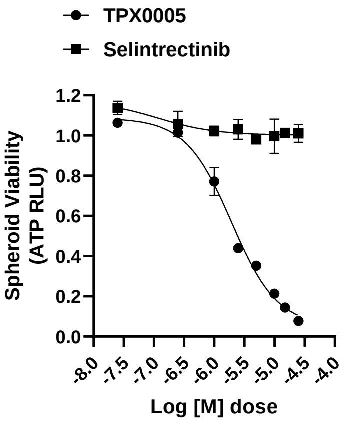

NTRK4 (Cell Adhesion Kinase, CAK) / DDR1 - i

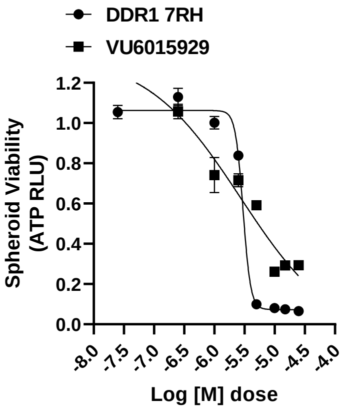

Developmental pathways-i

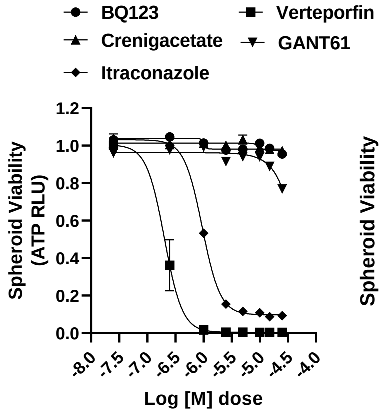

Epigenetic regulators - i

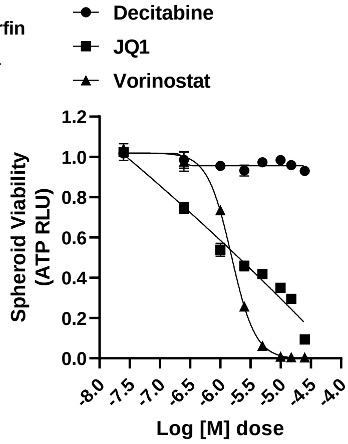

Multi-tyrosine kinase- I (DDR1/BCR-ABL)

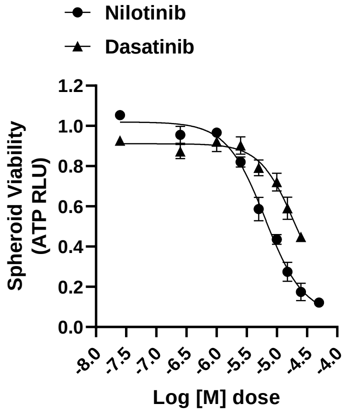

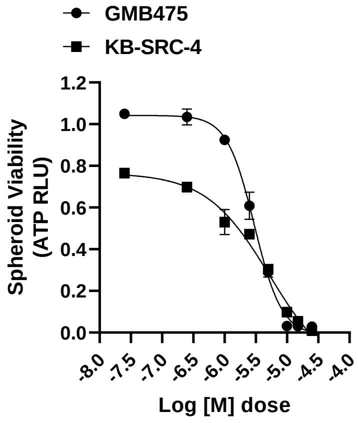

Supplementary Figure 3

(ii) HCT116 (cultured in media N2N21max)

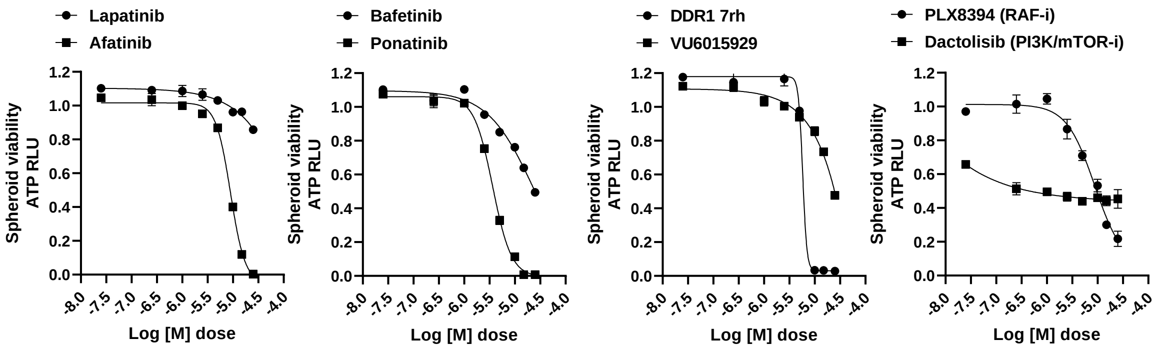

(iii) DLD1

RAF – i, PI3K/mTOR - i

Multi tyrosine kinase - i

NTRK4 (DDR1) - i

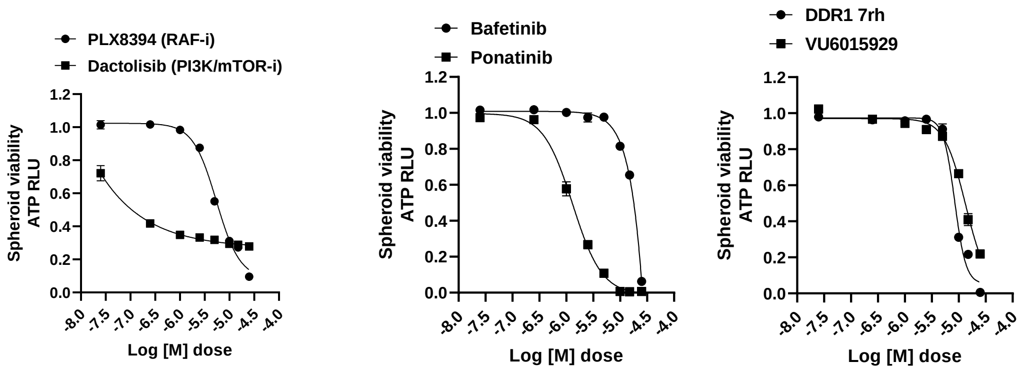

(iv) SW480

RAF – i, PI3K/mTOR - i

Multi tyrosine kinase - i

NTRK4 (DDR1)- i

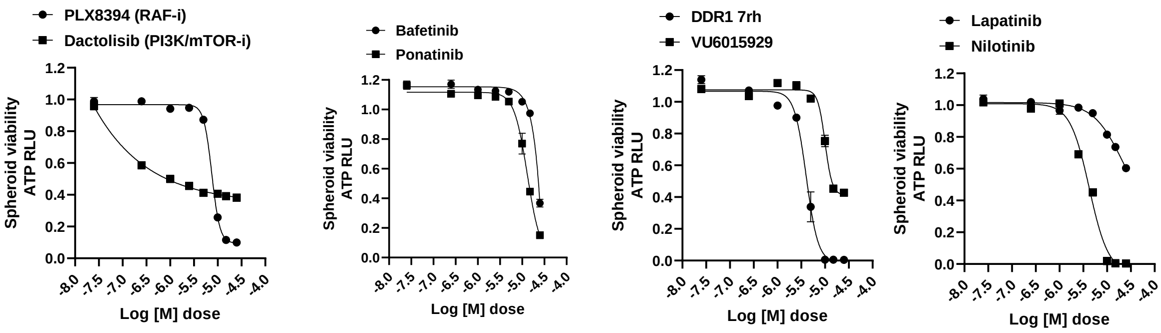

(B). Spheroid viability dose-response curves for Glioblastoma lines.

U251

Patient-derived GBM lines

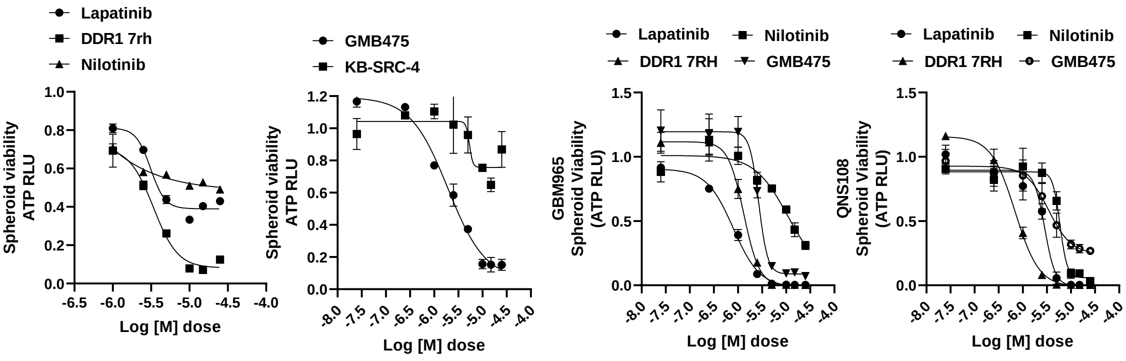

Supplementary Figure 3: (A). Spheroid viability curves for colorectal adenocarcinoma lines (i) HCT116 (cultured in media N2EF), (ii) HCT116 (cultured in media N2EF), (iii) DLD1, (iv) SW480, when administered with drugs as single agents, for each of inhibitor class indicated. **(B).** Spheroid viability curves for glioblastoma lines (U251, and Patient-derived GBM lines: GBM965 and QNS108) when administered with respective drugs as single agent.

(A).

Inhibitor combination plots of HCT116 (cultured in media N2EF).

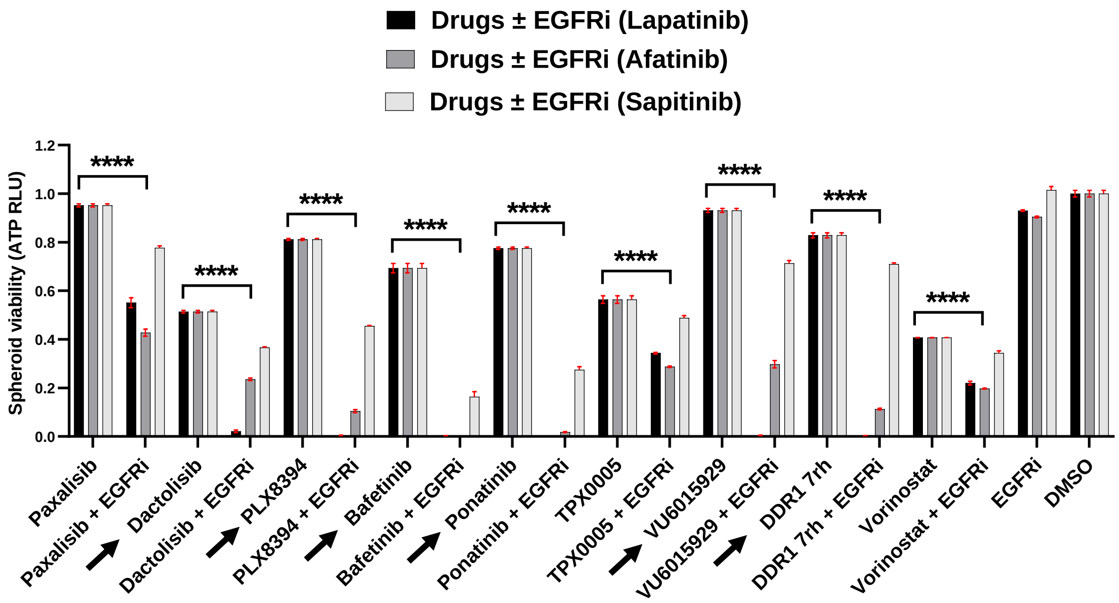

(B).

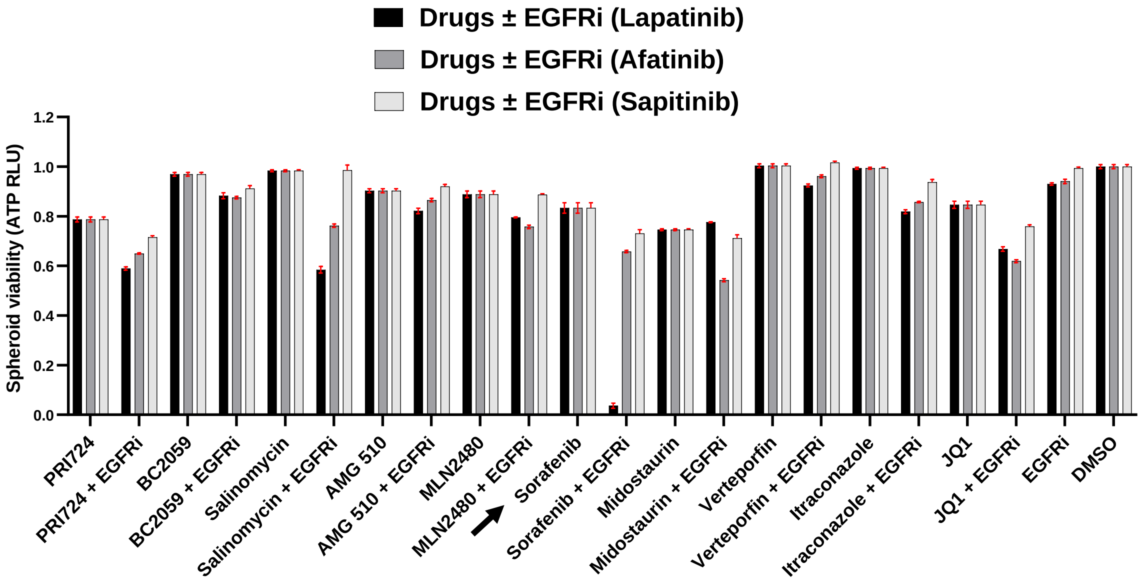

Supplementary Figure 4

Supplementary Figure 4: (A), (B). Bar-graphs showing spheroid viability for HCT116 (cultured in media N2EF) when administered with drugs as single agents, or in combinations at concentrations ≤ IC_{50}. Doses at which the drugs were combined, and Percent inhibition estimated has been included in Excel sheet 1.

(A).

Low High
Synergy Scores

Bafetinib (nM)

Lapatinib (nM)	0	3500	6500	13000	26000	37500
50000	0.0	69.8	69.0	51.5	5.9	2.3
28000	0.0	73.9	75.5	56.5	6.5	2.5
14000	0.0	71.6	85.6	64.2	7.2	2.7
7000	0.0	54.6	101.5	77.8	8.7	3.4
4800	0.0	51.2	94.0	75.6	8.4	3.3
0	0.0	0.0	0.0	0.0	0.0	0.0

Bafetinib (nM)

Afatinib (nM)	0	3500	6500	13000	26000	375000
50000	0	0	0	0	-0.047	-0.087
28000	0	0	0.051	0	-0.067	-0.024
14000	0	8.78	8.658	3.5113	-0.017	-0.044
7000	0	0	23.64	24.183	0	0
4800	0	0	23.22	27.128	0	0
0	0	0	0	0	0	0

Ponatinib (nM)

Lapatinib (nM)	0	250	500	1500	3000	6000
28000	0.0	16.2	39.4	52.2	48.6	21.0
14000	0.0	9.8	38.7	87.3	77.8	35.6
7000	0.0	13.3	17.5	85.8	93.3	43.0
2400	0.0	1.8	12.3	36.9	64.8	50.8
1200	0.0	0.0	0.8	5.1	27.0	50.0
0	0.0	0.0	0.0	0.0	0.0	0.0

Ponatinib (nM)

Afatinib (nM)	0	250	500	1500	3000	6000
28000	0	0	0.33	0.1442	0.137	0
14000	0	0	0.53	0.4871	0.29	-0.02
7000	0	0	0	62.061	39.94	1.67
2400	0	4.34	10.95	48.343	64.05	2.5942
1200	0	0	0	8.1662	61.62	2.6013
0	0	0	0	0	0	0

DDR1 7RH (nM)

Lapatinib (nM)	0	250	500	1500	3000	6000
28000	0.0	18.3	18.1	20.0	16.4	0.0
14000	0.0	36.7	51.1	52.1	40.4	0.2
7000	0.0	0.0	32.6	87.1	70.6	0.4
2400	0.0	0.0	0.0	11.9	93.2	0.0
1200	0.0	0.0	0.0	0.0	11.9	0.6
0	0.0	0.0	0.0	0.0	0.0	0.0

DDR1 7RH (nM)

Afatinib (nM)	0	250	500	1500	3000	6000
28000	0	0	0.244	0	0.047	0
14000	0	0	5.601	8.2434	7.343	4.11
7000	0	0	0	22.806	17.43	24.004
2400	0	0	0.379	3.2071	29.99	19.682
1200	0	0	0	0	0.609	0
0	0	0	0	0	0	0

VU6015929 (nM)

Lapatinib (nM)	0	500	1500	3000	6000	10000
15000	0.0	-10.7	0.0	0.0	63.1	47.3
10000	0.0	0.0	0.0	0.0	78.8	57.2
5000	0.0	0.0	0.0	0.0	67.1	56.4
2500	0.0	0.0	0.0	0.0	10.2	48.8
800	0.0	0.0	0.0	-1.8	0.0	29.1
0	0.0	0.0	0.0	0.0	0.0	0.0

VU6015929 (nM)

Afatinib (nM)	0	500	1500	3000	6000	10000
15000	0	0	0	0	1.236	7.5105
10000	0	0	0	0	10.43	0
5000	0	0	0	0	0	0
2500	0	1.87	0	0.943	6.984	0
800	0	10.1	1.153	0.6709	3.011	2.1701
0	0	0	0	0	0	0

PLX8394 (nM)

Lapatinib (nM)	0	500	1500	3000	6000	10000
15000	0	1.81	57.81	92.719	100.3	86.322
10000	0	2.76	12.83	59.893	96.64	85.687
5000	0	5.64	13.42	28.574	56.25	64.363
2500	0	0	10.28	25.438	34.26	32.936
800	0	0	0	11.94	17.8	12.451
0	0	0	0	0	0	0

PLX8394 (nM)

Afatinib (nM)	0	500	1500	3000	6000	10000
15000	0	0	1.151	0.188	0.003	0
10000	0	1.49	2.251	1.8913	0.748	0.5884
5000	0	25.8	49.07	61.065	41.13	34.68
2500	0	12.7	29.8	42.067	45.48	37.051
800	0	0.89	9.15	11.312	18.72	12.824
0	0	0	0	0	0	0

(B).

Low High
Percent inhibition

Bafetinib (nM)

Lapatinib (nM)	0	3500	6500	13000	26000	37500
50000	43.0	99.2	99.7	99.9	99.9	99.8
28000	37.1	98.1	99.7	99.8	99.9	99.8
14000	28.0	92.0	99.6	99.9	99.9	99.7
7000	13.3	73.5	98.2	99.9	99.8	99.7
4800	15.2	64.0	94.4	100.0	99.8	99.7
0	3.9	-7.8143	-4.843	22.441	91.155	96.123

Bafetinib (nM)

Afatinib (nM)	0	3500	6500	13000	26000	375000
50000	99.83	99.78	99.85	99.83	99.91	99.89
28000	99.77	99.90	99.94	99.88	99.90	99.91
14000	91.57	99.60	99.80	99.83	99.92	99.85
7000	38.82	58.30	87.42	99.82	99.90	99.83
4800	25.72	40.07	62.77	99.79	99.87	99.81
0	0.00	7.96	12.1	63.8	99.8	99.744

Ponatinib (nM)

Lapatinib (nM)	0	250	500	1500	3000	6000
28000	50.5	82.88	93.4	99.0	99.5	99.7
14000	23.8	47.127	64.4	99.6	99.7	99.8
7000	9.2	17.79	26.1	91.0	99.8	99.8
2400	-6.8	-5.8	1.1	30.0	75.3	98.5
1200	-5.0	-11.4	-5.7	5.6	36.7	85.1
0	0.0	-10.202	-12.94	-10.19	1.9109	54.0119

Ponatinib (nM)

Afatinib (nM)	0	250	500	1500	3000	6000
28000	99.65	99.54	99.95	99.84	99.93	99.92
14000	99.46	99.69	99.94	99.95	99.92	99.96
7000	41.29	33.69	43.18	99.86	99.92	99.95
2400	3.50	4.77	8.06	54.48	98.52	99.83
1200	3.74	-1.72	-0.43	24.38	96.09	99.88
0	0.00	-6.15	-8.38	-6.12	31.73	97.075

DDR1 7RH (nM)

Lapatinib (nM)	0	250	500	1500	3000	6000
28000	81.5	98.8	99.5	99.7	99.8	99.8
14000	53.1	91.5	99.7	99.9	99.6	99.9
7000	21.7	35.6	53.0	99.9	99.9	99.9
2400	-3.4	-0.7	1.6	24.9	99.9	99.6
1200	-4.9	-9.2	-8.4	-3.6	34.3	99.8
0	0.0	-11.9	-10.5	-11.3	9.8	99.2

DDR1 7RH (nM)

Afatinib (nM)	0	250	500	1500	3000	6000
28000	99.58	99.47	99.87	99.67	99.74	99.77
14000	89.90	89.00	95.78	98.33	99.36	99.82
7000	42.40	36.76	33.64	67.85	84.19	99.72
2400	5.25	7.44	9.26	12.09	60.57	90.27
1200	4.88	-0.20	0.63	6.64	42.54	69.42
0	0.00	-5.47	-5.24	-2.59	19.75	57.931

VU6015929 (nM)

Lapatinib (nM)	0	500	1500	3000	6000	10000
15000	28.8	14.2	17.0	56.3	99.2	97.6
10000	13.3	13.7	7.0	45.2	99.0	96.0
5000	5.3	7.7	11.4	10.0	86.7	91.7
2500	0.1	4.8	7.4	0.0	26.9	81.6
800	1.1	1.7	3.1	-8.6	14.6	62.9
0	0.0	5.62	4.57	2.14	9.81	29.83

VU6015929 (nM)

Afatinib (nM)	0	500	1500	3000	6000	10000
15000	78.01	54.96	83.71	81.93	89.14	92.79
10000	25.36	27.00	30.77	37.69	40.00	42.42
5000	8.66	8.18	13.31	13.75	15.58	14.08
2500	-2.92	4.12	5.45	5.50	7.06	10.94
800	-10.85	0.65	-3.05	-0.01	-0.03	2.08
0	0.00	-14.8	-11.4	-6.16	-8.53	-8.286

PLX8394 (nM)

Lapatinib (nM)	0	500	1500	3000	6000	10000
15000	3.2	12.9	48.7	81.6	92.3	93.1
10000	-1.4	7.5	16.0	58.0	87.0	87.6
5000	-7.7	-2.6	5.8	14.0	50.1	68.0
2500	-11.5	-8.7	-3.9	-1.2	15.2	35.2
800	-13.1	-15.7	-9.7	-13.2	0.6	12.0
0	0.0	-19.6	-19.3	-18.9	-11.6	1.5

PLX8394 (nM)

Afatinib (nM)	0	500	1500	3000	6000	10000
15000	98.78	98.55	99.87	99.43	99.65	99.56
10000	97.77	99.19	99.79	99.86	99.84	99.85
5000	9.84	27.20	73.17	90.84	99.64	99.81
2500	-8.83	-1.37	20.92	62.61	95.93	97.30
800	-9.80	-9.16	4.72	42.28	73.40	82.09
0	0.00	-17.4	-10.2	8.2	55.08	62.911

Supplementary Figure 5

(C). Dot plots and Spheroid viability graphs for Synergy testing in HCT116.

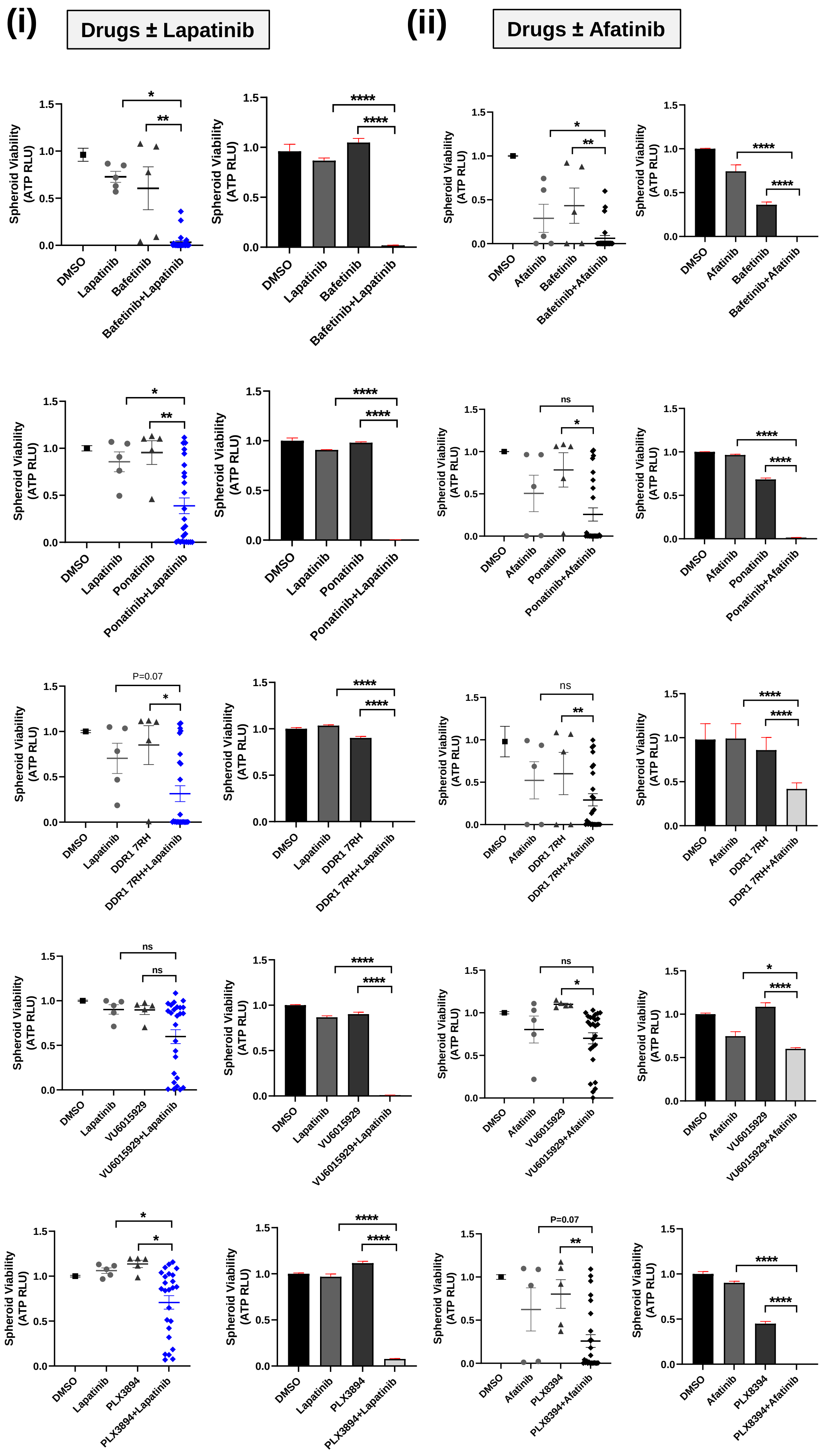

Table 2: Synergy scores obtained in HCT116 multispheroid viability assay.

		Cumulative scores obtained		Max. Synergy obtained at:		
**Drug 1**	**Drug 2**	**Synergy Score**	**Antagonism**	**Drug 1**	**Drug 2**	**Synergy score**
Lapatinib	Bafetinib	1123.4	0	7µM	6.5µM	101.45
	Ponatinib	928.87	0	7µM	3µM	93.3
	DDR1 7rh	561.5	0	2.4µM	3µM	93.2
	VU6015929	458.13	-12.44	10µM	6µM	78.78
	PLX8394	910.15	0	15µM	6µM	100.3
Afatinib	Bafetinib	119.18	-0.29	4.8µM	13µM	27.13
	Ponatinib	308.25	-0.02	2.4µM	3µM	64.04
	DDR1 7rh	143.69	0	2.4µM	3µM	30
	VU6015929	46.05	0	10µM	6µM	10.43
	PLX8394	439.98	0	5µM	3µM	61.06

(D).

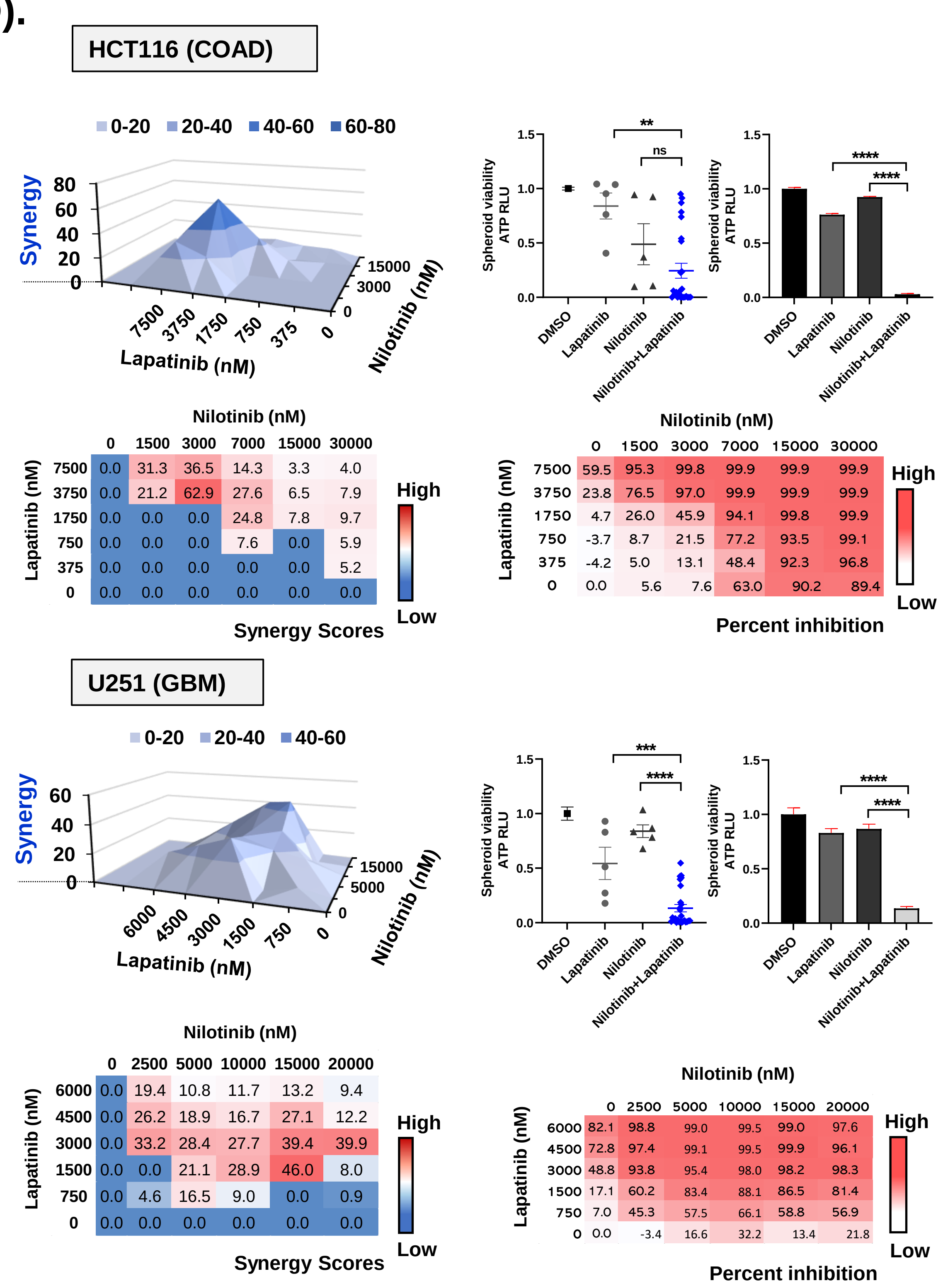

Cell line	Drug 1	Drug 2	Cumulative scores obtained: Synergy Score	Antagonism	Max. Synergy obtained at: Drug 1	Drug 2	Synergy score
**HCT116**	Lapatinib	Nilotinib	276.57	0	3.75µM	3µM	62.9
**U251**	Lapatinib	Nilotinib	469.3	0	1.5µM	15µM	45.96

Supplementary Figure 5: (A), (B). Matrix of synergy score values and a matrix of percent inhibition obtained for all 25 combination treatments in each of the respective synergy experiment performed. **(C).** Dot plots showing relative spheroid viability obtained for all 5 doses of each drug (administered as single agent), and for all 25 combination treatments done. Bar-graphs, showing the relative spheroid viability at combinatorial doses where synergy obtained was maximum. Table-2 states the cumulative synergy- and antagonism-scores obtained for all combinations tested (drugs with Lapatinib or Afatinib), the doses of drug1 and drug2 at which synergy was maximum, and maximum synergy score value for each of the combination tested (software used: MacSynergy-II). **(D).** Synergy plots for Lapatinib ± Nilotinib, for HCT116 multi-spheroids cultured in N2EF media, and U251 multi-spheroids. Table (bottom) states the cumulative synergy- and antagonism- scores obtained. *p < 0.05, **p < 0.01, ***p < 0.001, ****p < 0.0001.

(A).

Correlation analysis (Normal vs. Tumor)													
S.No.	Gene	Normal (Colon)	Tumor (COAD)	Normal (Brain Cortex)	Tumor (GBM)	PanCancer Atlas		Normal (Colon)	Tumor (COAD)	Normal (Brain Cortex)	Tumor GBM	PanCancer Atlas	
		DDR1						KRAS					
		R_P	R_P	R_P	R_P	R_P	R_S	R_P	R_P	R_P	R_P	R_P	R_S
1	EGFR	-0.23	0.36	0.38	0.41	0.25	0.35	0.18	0.18	-0.06	0.15	0	0.16
2	IGF1R	-0.68	0.34	0.18	0.14	0.03	0.23	-0.23	0.2	0.48	0.34	0	0.04
3	ERBB2	0.91	0.12	0.66	0.43	0.04	0.29	0.59	0.022	-0.12	0.18	0.02	0.11
4	ERBB3	0.89	0.51	0.63	0.049	-0.1	0.01	0.54	0.081	-0.13	0.13	0.05	0.12
5	ERBB4	-0.27	-0.045	-0.23	0.39	0.24	0.31	-0.13	0.062	0.7	0.41	0.01	0.04
6	KRAS	0.74	0.2	-0.26	0.32	0.01	0.12	1	1	1	1	1	1
7	BRAF	-0.67	0.25	-0.11	0.36	0.13	0.21	-0.37	0.24	0.89	0.56	0.11	0.46
8	PIK3CA	-0.55	0.33	-0.019	0.24	-0.05	-0.01	-0.15	0.34	0.71	0.49	0.11	0.16
9	MTOR	-0.57	0.21	0.084	0.52	0.02	0.08	-0.31	0.22	0.52	0.49	0.08	0.32
10	SRC	0.48	0.33	-0.077	0.2	0.04	0.14	0.4	0.22	0.6	0.38	0.06	0.13
11	LYN	0.29	0.1	0.25	0.03	-0.15	-0.09	0.44	0.14	0.043	0.18	0.07	0.22
12	BCR	-0.0056	0.24	0.0029	0.43	0.43	0.4	0.24	0.17	0.38	0.37	-0.02	0.05
13	ABL1	-0.66	0.36	0.79	0.37	0.07	0.11	-0.32	0.34	-0.043	0.51	-0.05	-0.06
14	ABL2	-0.47	0.15	0.0041	0.033	-0.09	-0.09	-0.13	0.19	0.77	0.29	0	0.23
15	SOX2	-0.57	0.025	0.53	0.56	0.48	0.09	-0.32	-0.029	-0.1	0.45	0	0.23
16	SOX5	-0.56	0.025	0.1	0.3	0.58	0.31	-0.24	0.086	0.42	0.59	0.01	0.18
17	SOX7	-0.37	-0.11	0.27	0.18	0.17	0	-0.094	0.082	0.085	0.69	-0.01	0.01
18	SOX8	-0.36	0.26	0.7	0.19	0.01	0.02	-0.27	-0.0011	-0.15	0.35	0.02	0.07
19	SOX9	0.84	0.22	0.55	0.66	0.49	0.12	0.68	0.097	-0.22	0.4	-0.04	-0.18
20	SOX10	-0.53	0.0013	0.68	-0.01	0.29	0.34	-0.36	0.073	-0.18	0.084	0.05	0.12
21	POU5F1	0.51	0.17	-0.0034	0.061	-0.08	0.06	0.25	-0.035	0.17	0.01	-0.08	-0.04
22	VANGL2	-0.52	0.26	0.69	0.59	-0.09	-0.06	-0.22	0.26	0.023	0.45	0.12	0.15
23	LGR5	0.71	0.028	0.55	-0.14	0.46	0.43	0.58	-0.0098	-0.026	0.081	0.04	0.11
24	APC	-0.15	0.25	-0.11	0.51	-0.03	0.14	0.22	0.39	0.58	0.61	0.03	0.23
25	CTNNB1	0.25	0.2	0.089	0.37	-0.03	0.09	0.62	0.079	0.76	0.41	0.03	0.17
26	CTNNA1	0.25	0.1	0.54	0.66	0.08	0.18	0.51	0.29	0.43	0.45	0.05	0.16
27	CDH1	0.93	0.45	0.51	0.35	0.07	0.25	0.81	0.26	-0.064	0.26	0.05	0.19
28	CDH2	-0.31	-0.061	-0.14	0.49	0.16	-0.02	-0.42	0.087	-0.0027	0.4	-0.04	-0.19

(B).

Colon (normal)

	EGFR	DDR1	SRC	LYN	BCR	CDH1	LGR5	
EGFR	1	3.30E-05	0.15	0.84	0	2.00E-04	0.002	EGFR
DDR1	-0.23	1	0	0.31	1.60E-06	0	0	DDR1
SRC	0.082	0.48	1	0.17	0.56	1.80E-15	0	SRC
LYN	-0.011	0.058	-0.078	1	0.027	0.092	0.31	LYN
BCR	0.47	-0.27	0.033	0.13	1	9.30E-07	2.30E-01	BCR
CDH1	-0.21	0.93	0.43	0.096	-0.28	1	0	CDH1
LGR5	-0.18	0.71	0.48	0.058	0.064	6.80E-01	1	LGR5

COAD

	EGFR	DDR1	SRC	LYN	BCR	CDH1	LGR5	
EGFR	1	1.3E-09	6.8E-06	0.05	0.000005	0.000002	0.68	EGFR
DDR1	0.36	1	1.9E-08	0.097	0.000066	1.8E-15	0.64	DDR1
SRC	0.27	0.33	1	0.15	0.000044	0	0.64	SRC
LYN	0.12	0.1	0.087	1	1.2E-11	0.35	0.17	LYN
BCR	0.27	0.24	0.24	0.39	1	0.000017	0.77	BCR
CDH1	0.28	0.45	0.61	-0.057	0.26	1	0.015	CDH1
LGR5	-0.025	0.028	0.029	-0.083	-0.018	0.15	1	LGR5

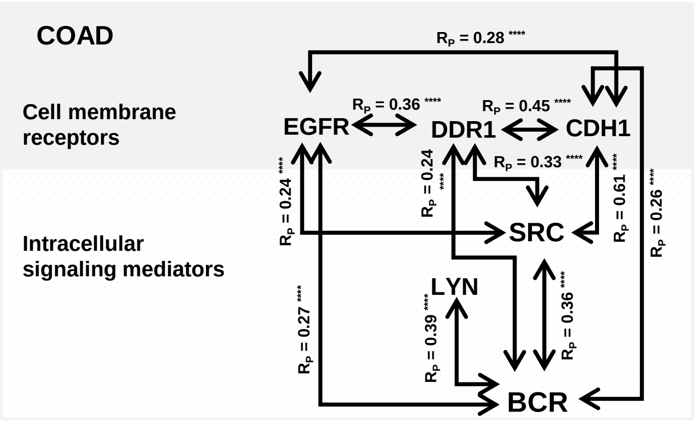

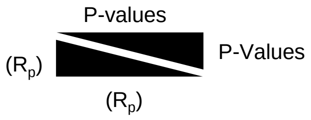

Supplementary Figure 6

(C).

STRING database
Network: Physical interactions
Confidence level: High (score > 0.7)
MCL clustering (Inflation: 5)
Number of Nodes: 32
Number of edges: 112
PPI enrichment p-value: <1.0e-16
Edges within a cluster: Solid line
Edges between clusters: Dotted line

Clusters	Proteins
Cluster 1	APC,AXIN1,CDH1,CTNNA1,CTNNB1,GSK3B,SOX2,SOX9
Cluster 2	ABL1,BCR,GAB1,GRB2,IRS1,LYN,SOS1
Cluster 3	EGF,EGFR,ERBB2,ERBB3,EREG,TGFA
Cluster 4	DDR1,PTPN11,SHC1
Cluster 5	DVL2,VANGL2
Cluster 6	BRAF,KRAS
Cluster 7	PIK3CA,SRC
Cluster 8	AKT1

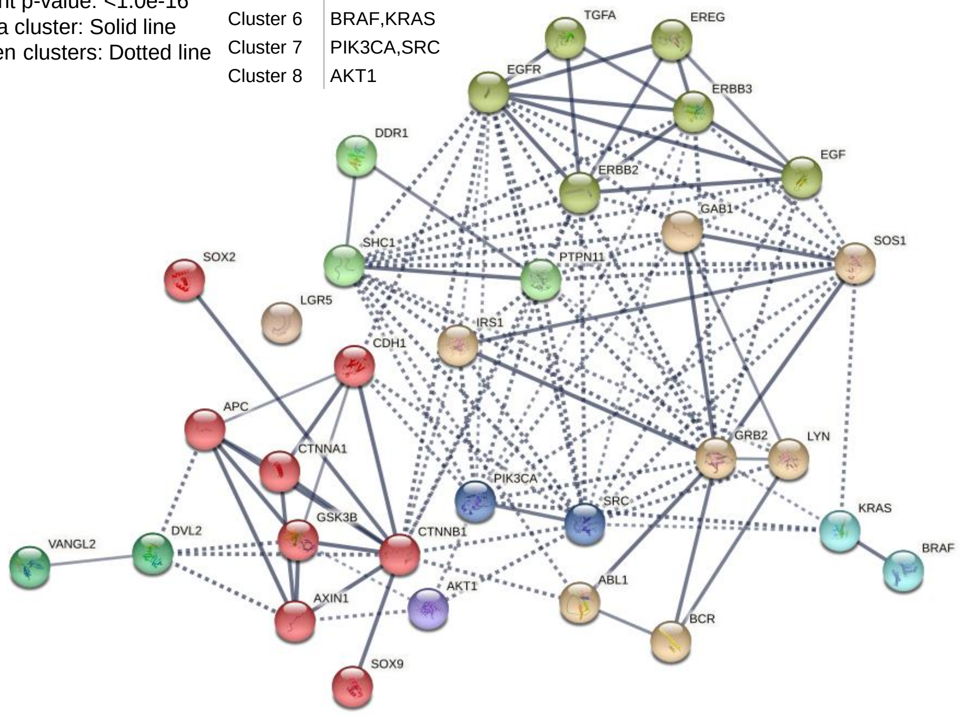

(D).

Gene	COAD		GBM	
	Normal	Tumor	Normal	Tumor
EGFR	3.6	3.1	2.3	6.4
→ ERBB2	5.1	6.1	2.6	4.3
→ DDR1	5.1	6.8	5.6	8.4
DDR2	5.2	1.9	1.9	3.2
→ BCR	4.2	4.9	5.4	5.1
ABL1	5.9	4.8	3.8	5
SRC	5.5	5.9	4	4.1
→ LYN	2.7	4.5	1.8	4.2
→ LGR5	0.6	3.9	0.8	0.5
→ CTNNB1	6.5	7.6	5.7	7.5
APC	2.5	2	4.6	3.9
PDGFRA	4.8	2.7	2.9	4.4
FGFR1	6.6	3.3	4.4	5.5
VEGFR2	2.3	2.1	1.2	2.8

Scale: Log_2 (TPM + 1): 10, 9, 8, 7, 6, 5, 4, 3, 2, 1, 0

Genes upregulated in tumors	
COAD	ERBB2, DDR1, BCR, SRC, LYN, LGR5, CTNNB1
GBM	EGFR, ERBB2, DDR1, DDR2, ABL1, LYN, CTNNB1, PDGFRA, FGFR1, VEGFR2

(E).

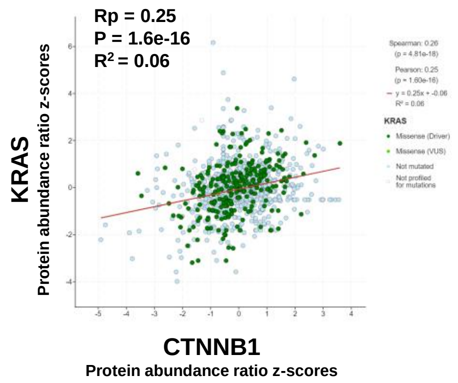

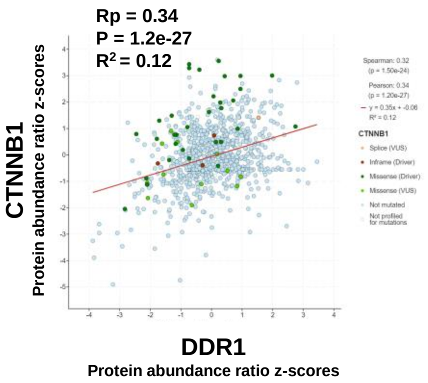

Figure 5: (A). Genomic associations of gene DDR1 with genes relating to other targets of the combinatorial (top-5) drug-leads identified. Datasets evaluated for tumors vs. normal, include colorectal adenocarcinoma (COAD), Glioblastoma (GBM), Colon (normal) and Brian cortex (normal) utilizing GEPIA; and Pan-Cancer Atlas using cBioPortal. All correlation coefficient values included are Pearson (Rp), except for TCGA pan-cancer atlas for which both Rp (Pearson) and Rs (Spearman) values were considered here due to intrinsic variability and heterogeneity among various tumor types. **(B).** Correlation matrix represents correlation coefficients, R-Pearson (Rp) obtained between the mRNA expression of each of the genes indicated on y-axis and the genes on x-axis (Source: GEPIA). (**C).** DDR1/BCR-ABL signaling networks using STRING database: The network (32 nodes and 112 edges), includes proteins reported to be engaging in physical interactions with high confidence (score > 0.7). Clustering performed utilizing MCL method revealed eight clusters listed at the top of the protein network. The clustering indicates SHC1 to be the adaptor protein that links DDR1 to other signaling pathways, including EGFR/ERBB2 and BCR-ABL complex which activate KRAS downstream; Interaction sources utilized in STRING database involved: Textmining, Experiments, Co-expression, Databases, Neighborhood, Co-occurrence (but, excluding gene-fusions). **(D).** Transcriptional expression values obtained using GEPIA for selective genes in datasets of COAD and GBM (Tumor vs. normal) represented in scale: Log_2 (TPM + 1). **(E).** Correlation between protein levels of CTNNB1 (beta-catenin), KRAS, and DDR1 in patient datasets available from curated non-redundant studies including TCGA Pan Cancer deposited at cBioPortal (graphs show ~ 950 patient samples from 202 independent studies) .

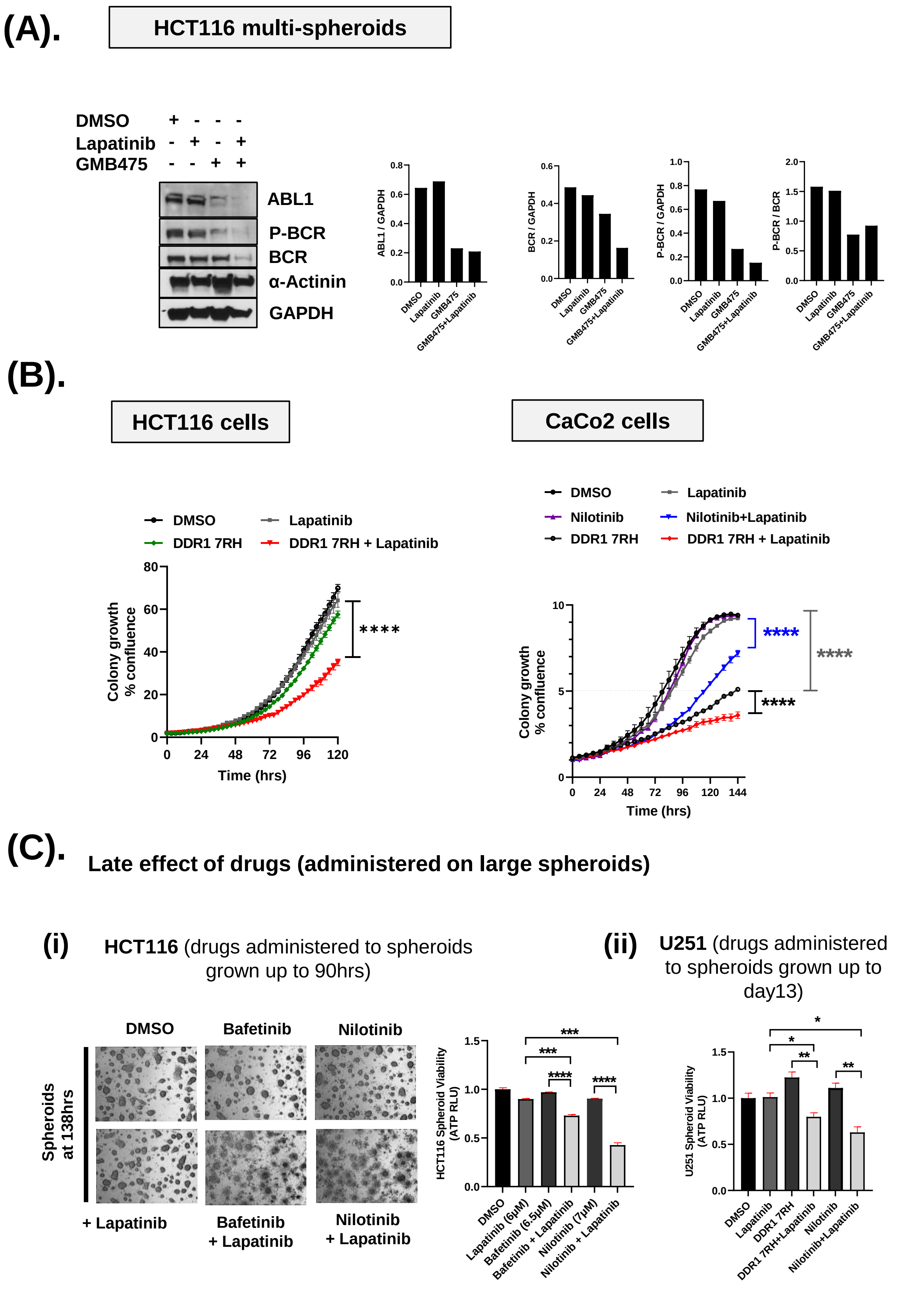

Supplementary Figure 7

Supplementary Figure 7: (A). Western blots on HCT116 multi-spheroid lysates show reduction in levels of ABL1 protein and phospho-BCR upon administration of ABL1 PROTAC-inhibitor, GMB475, at 24hr post-treatment. Total BCR levels were also reduced upon combination treatment indicating that Lapatinib in combination with GMB475 at early time-points may also impact BCR gene regulation thereby altering not only signaling but also over all protein content in the cells. Actinin and GAPDH were used as loading controls. **(B).** Clonal cell proliferation assay performed using Incucyte for HCT116 and CaCo2 for drugs indicated. Nilotinib had better efficacy, when administered in combination with Lapatinib. DDR1 inhibition by 7RH drug was significantly cytotoxic both as single agent and in combination. P-values are indicated, $*p < 0.05$, $**p < 0.01$, $***p < 0.001$, $****p < 0.0001$. **(C).** Evaluation of late effects of drug treatment, by having the inhibitors administered on large (over-sized) spheroids. (i) HCT116 spheroids were cultured for 2 days, images were acquired at 48hrs, and then, continued to be grown till 90hrs (~day4), when drugs were administered. Spheroids were reimaged at 138hrs (~ day6), and then lysed for evaluation of viability. Bar-graph below shows significant reduction in combinations as compared to drugs administered alone. (ii) U251 spheroids were cultured till day 13, drugs were administered, and then, effect of drugs on spheroid viability was estimated at day15. Bar-graph shows significant reduction in combinations as compared to drugs administered alone (bar-graph).

(A).

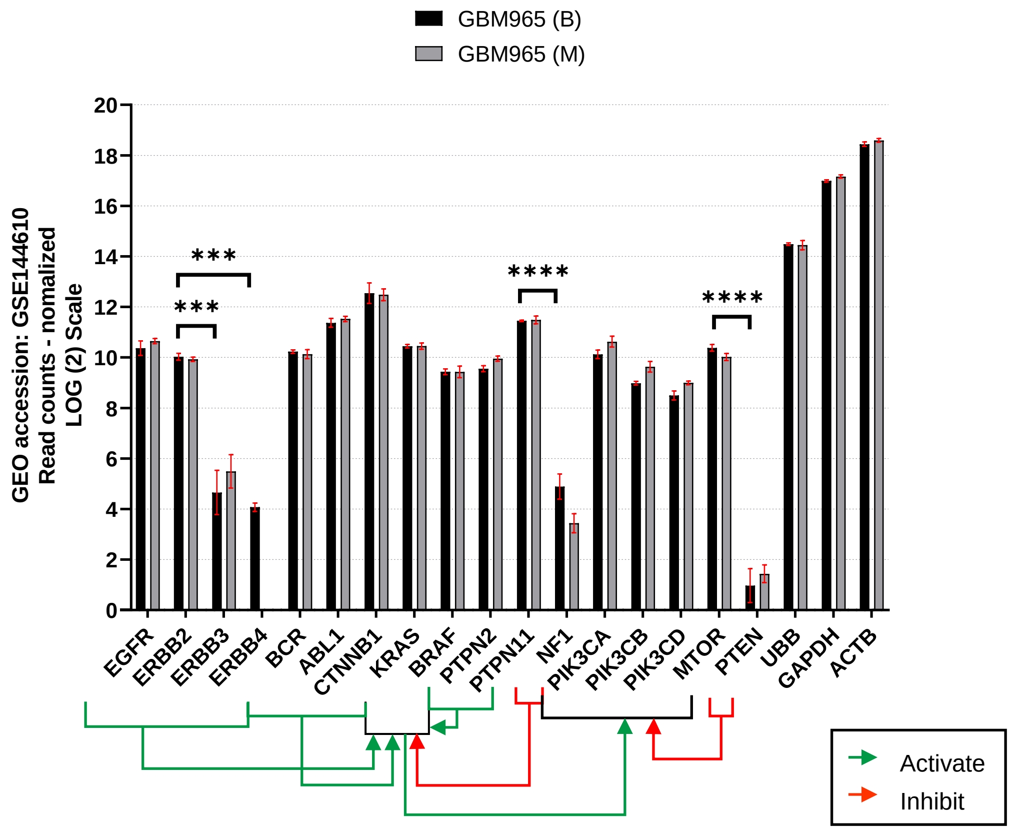

(B). **Fold difference in read counts (normalized)**

	GBM965 (B)	GBM965 (M)
EGFR	3.26	3.82
ERBB2	2.49	2.31
ERBB3	0.09	0.13
ERBB4	0.03	0.00
BCR	2.85	2.69
ABL1	6.35	7.00
CTNNB1	15.37	13.87
KRAS	3.31	3.34
BRAF	1.65	1.67
PTPN2	1.79	2.36
PTPN11	6.61	6.85
NF1	0.08	0.03
PIK3CA	2.67	3.82
PIK3CB	1.20	1.92
PIK3CD	0.87	1.21
MTOR	3.17	2.48
PTEN	0.01	0.01
UBB	54.19	53.75
GAPDH	307.28	347.61
ACTB	844.13	937.11

EGFR/ERBB2 inhibitor (Lapatinib)

DDR1/BCR-ABL1 MTK inhibitors (Nilotinib)

PIK3/MTOR dual kinase inhibitors (Dactolisib, Paxalisib)

Fold change >1 (Fold increase in average counts)

Fold change < 1 (Fold decrease in average counts)

Fold change = 1 (Average read counts = 433)

Supplementary Figure 8

Supplementary Figure 8: (A). Bar-graphs showing normalized read counts (Scale: Log_2) for patient derived GBM line, GBM965 (Gene expression dataset used: GSE144610). The transcriptional expression of proteins that positively activate KRAS (EGFR/ERBB2, BCR-ABL1, CTNNB1, PTPN2, PTPN11) were upregulated, while the negative regulator of KRAS (NF1) had lower expression. Additionally, KRAS downstream targets like, BRAF, PIK3CA, MTOR were upregulated, and the negative regulator of PIK3/AKT pathway, the tumor suppressor PTEN had lower expression reads (***$p < 0.001$, ****$p < 0.0001$). **(B).** Fold difference between reads (counts-normalized) for every gene with respect to the average number of transcriptional reads (average counts-normalized) observed in the entire dataset. The data is presented along with expression values of three house-keeping genes (ACTB, beta-actin; Glyceraldehyde 3-phosphate dehydrogenase, GAPDH; Ubiquitin, UBB). Key: GBM965 (B), GBM965 unsorted bulk cells; GBM965 (M), GBM965 sorted for having migratory potential.
